# The Roles and Mechanisms of lncRNAs in Liver Fibrosis

**DOI:** 10.3389/fphar.2021.779606

**Published:** 2021-11-24

**Authors:** Zhifa Wang, Xiaoke Yang, Siyu Gui, Fan Yang, Zhuo Cao, Rong Cheng, Xiaowei Xia, Chuanying Li

**Affiliations:** ^1^ Department of Rehabilitation Medicine, Chaohu Hospital of Anhui Medical University, Hefei Anhui, China; ^2^ Department of Rheumatology and Immunology, The First Affiliated Hospital of Anhui Medical University, Hefei, China; ^3^ Department of Ophthalmology, The Second Affiliated Hospital of Anhui Medical University, Hefei, China; ^4^ The First Clinical Medical College, Anhui Medical University, Hefei, China; ^5^ Department of Gastroenterology, Anhui Provincial Children’s Hospital, Hefei, China

**Keywords:** lncRNAs, liver fibrosis, ceRNAs, HSCs, therapeutic strategies

## Abstract

Long non-coding RNAs (lncRNAs) can potentially regulate all aspects of cellular activity including differentiation and development, metabolism, proliferation, apoptosis, and activation, and benefited from advances in transcriptomic and genomic research techniques and database management technologies, its functions and mechanisms in physiological and pathological states have been widely reported. Liver fibrosis is typically characterized by a reversible wound healing response, often accompanied by an excessive accumulation of extracellular matrix. In recent years, a range of lncRNAs have been investigated and found to be involved in several cellular-level regulatory processes as competing endogenous RNAs (ceRNAs) that play an important role in the development of liver fibrosis. A variety of lncRNAs have also been shown to contribute to the altered cell cycle, proliferation profile associated with the accelerated development of liver fibrosis. This review aims to discuss the functions and mechanisms of lncRNAs in the development and regression of liver fibrosis, to explore the major lncRNAs involved in the signaling pathways regulating liver fibrosis, to elucidate the mechanisms mediated by lncRNA dysregulation and to provide new diagnostic and therapeutic strategies for liver fibrosis.

## 1 Introduction

### 1.1 Overview of Liver Fibrosis

**FIGURE 1:**
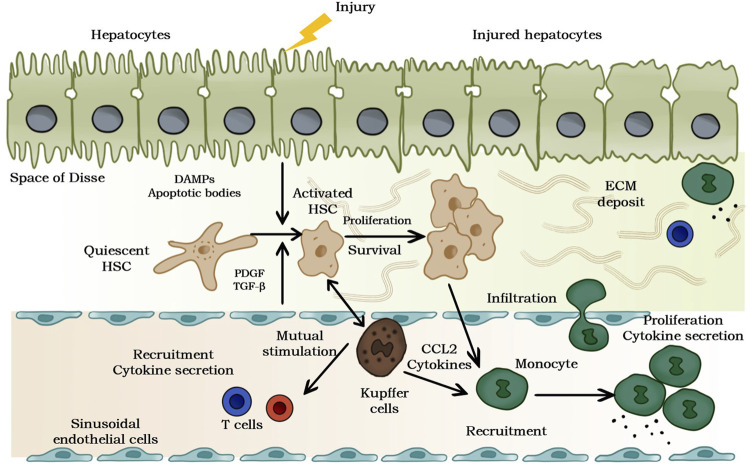
Pathogenesis of liver fibrosis. The release of damage-related patterns (DAMPs). and apoptotic bodies can be induced by chronic hepatocyte injury, which activates hematopoietic stem cells and recruits immune cells. Moreover, the complex multidirectional interaction between activated hematopoietic stem cells and Kupffer cells and innate immune cells promotes transformation and differentiation into proliferation and ECM to generate myofibroblasts.

As a globally important public health problem, liver fibrosis is typically characterized by a reversible wound healing response and an accompanying imbalance between increased synthesis and deposition and decreased degradation of extracellular matrix (ECM), resulting in programmed overaccumulation of ECM components (Nudelman et al., 1998; Aydin and Akcali, 2018). Numerous epidemiological studies have revealed the etiological role of various chronic liver diseases and associated liver injury-healing reactions in liver fibrosis, such as hepatitis (non-alcoholic steatohepatitis (NASH), hepatitis B and C and so on) and biliary obstruction, which are closely associated with its progression (Parola and Pinzani, 2019). Mechanistic studies at the cellular level suggest that hepatic stellate cells (HSCs) located in the Disse space between hepatic sinusoidal endothelial cells and hepatic epithelial cells and maintaining a close interaction with both are the main sites for the production of ECM components (Geerts, 2001; Khomich et al., 2019), and furthermore, numerous studies have revealed that their intracellular lipid droplets, which are specific organelles for hepatic retinoic acid storage (Blaner et al., 2009; Elpek, 2014), could lead to liver disease disorders through efflux, depletion, and loss. undesirable progression (Yin et al., 2013; Ray, 2014; Krizhanovsky et al., 2008). Thus, studies on the activation mechanisms of hematopoietic stem cells are of great concern in proposing new therapies against hepatic fibrosis and in improving the original strategies (Figure 1).

### 1.2 Overview of LncRNAs

In recent years, numerous non-coding RNAs (ncRNAs) molecules have been identified benefiting from the application of RNA microarrays and next-generation transcriptome sequencing technologies, enabling humans to deepen their understanding of the pathophysiology of multiple diseases from a new perspective (Consortium et al., 2007). ncRNAs are well known for not encoding proteins at the RNA level but can perform as key regulators of multiple regulatory gene expression as well as cellular signaling pathways (Heo et al., 2019). NcRNAs are categorized according to their relative size into two types: small or short non-coding RNAs (miRNAs) of less than 200 nucleotides (nt) and long non-coding RNAs (lncRNAs) of greater than 200 nucleotides (Riaz and Li, 2019). The most prominently researched endogenous small ncRNAs, known as miRNAs, mainly regulate the post-transcriptional levels of target genes by binding to the 3′ untranslated region (3′ UTR) of mRNAs, thus playing an important role in regulating the cell growth cycle as well as the expression of specific cell differentiation and cell death-related genes, lipid metabolism, and inflammatory responses. miRNAs have shown association with various liver diseases including liver fibrosis (Zhang CY. et al., 2016; Lan et al., 2018; Zhao et al., 2019).

**FIGURE 2:**
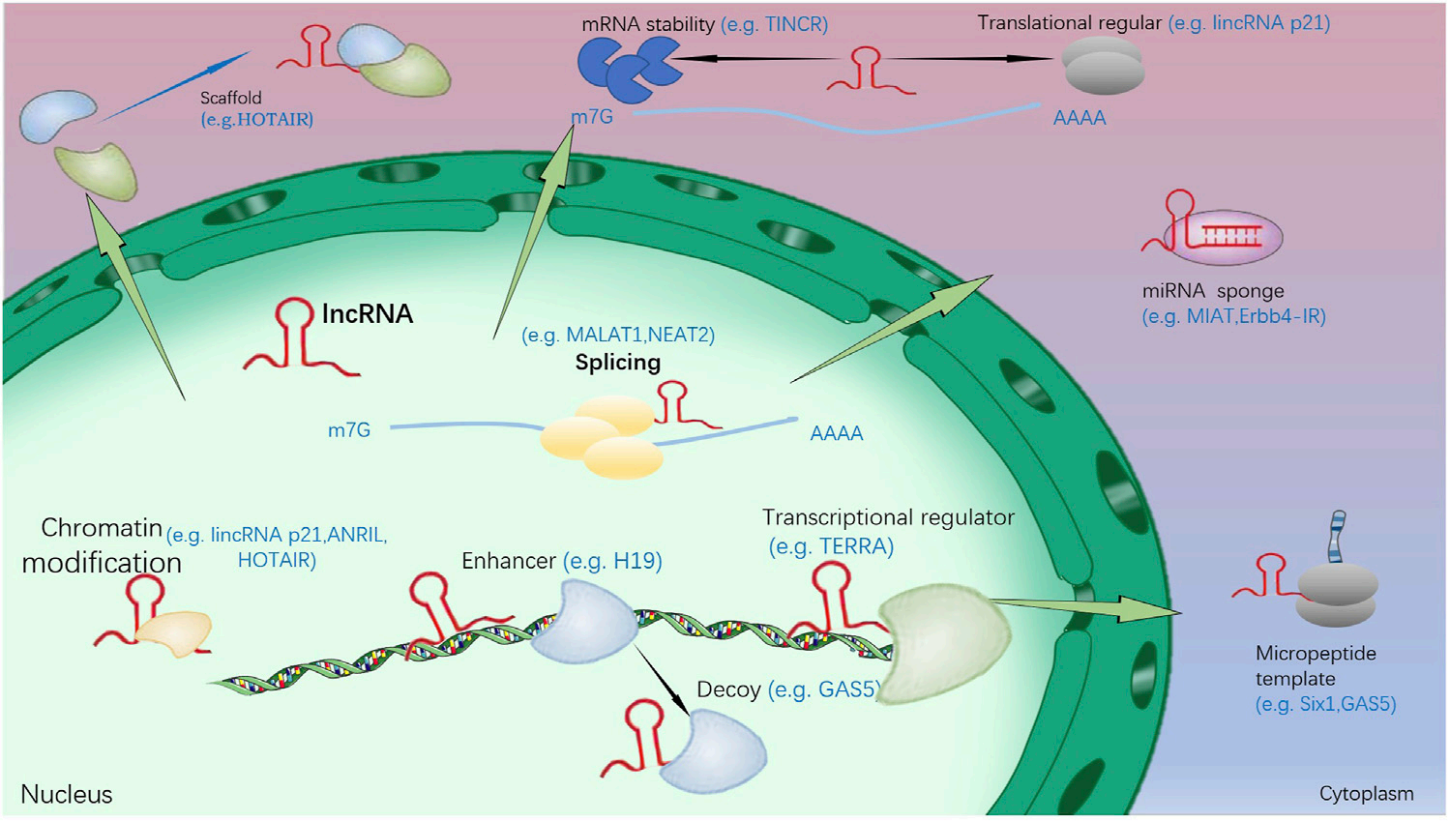
The function and regulation mechanism of lncRNA. (1): In the nucleus, lncRNA could inhibit and/or activate gene expression by transferring chromatin modifiers and various transcriptional regulators into DNA. In addition, target gene activation could be further enhanced by lncRNA. They can also induce proteins to move away from specific DNA locations and pass as molecular decoys. (2): In the cytoplasm, lncRNA could bring two or more proteins into a complex by acting as a scaffold. In addition, they could regulate other transcripts or proteins by acting as sponges and protein templates, or regulating mRNA degradation and translation.

As a novel ncRNAs, lncRNAs are predominantly transcribed by RNA polymerase II and exhibit multiple functions at the molecular level (Figure 2) (Ma et al., 2016b), the lncRNAs are classified according to their relative position on the chromosome to the coding gene as: 1. antisense lncRNAs, 2. intronic lncRNAs, 3. divergent lncRNAs, 4. intergenic lncRNAs, 5. promoter upstream lncRNAs, 6. promter-associated lncRNAs, 7. transcription start site-associated lncRNAs. LncRNAs regulate the expression of different genes based on their different cellular locations in multiple molecular mechanistic pathways including chromatin modification, transcriptional regulation, and post-transcriptional regulation (Zhang et al., 2014; Kopp and Mendell, 2018).

#### 1.2.1 LncRNA Regulates DNA Methylation Modifications

Tsix inhibits Xist transcription by recruiting CTCF to the Xist promoter region, while JPx inhibits CTCF transcriptional repression of Xist by binding CTCC (CCCTC-binding factor); Khps1a participates in the T-DMR (tissue-dependent) region demethylation of Sphk1 by an unknown mechanism; Dum *cis*-recruits DNMT1, DNMT3A, DNMT3B, and etc. to the promoter region of the neighboring gene Dppa2. differentially methylated region) of Sphk1; Dum *cis*-recruits DNMT1, DNMT3A and DNMT3B to the promoter region of the neighboring gene Dppa2 and causes silencing of methylation in this region, thereby suppressing Dppa2 expression and leading to differentiation of skeletal muscle myogenic cells into myoblasts (Guttman et al., 2011; Bian et al., 2019; He et al., 2020).

#### 1.2.2 LncRNAs Are Involved in Pre-transcriptional Regulation

Xist (X chromosome inactivation specific transcript) and RepA (transcript of the adenine repeat region at the 5′ end of the Xist gene) synergistically wrap the X chromosome and recruit PRC2 to establish H3K27me3 to cause X chromosome inactivation; Bxd (Bithoraxoid) binds to ubx-TRE (Ubx *cis*-regulatory trithorax response elements) and recruits ASH1 to activate Ubx transcription; HOTAIR regulates the expression of the HoxD gene cluster in trans by interacting with two histone modification complexes: catalytic PRC2 complex established by H3K27me3, and the LSD1-CoREST-REST complex catalyzing H3K4me2/3 erasure (lysine-specific demethylase1-RE1-Silencing Transcription factor corepressor 1-RE1 -Silencing Transcription factor complex); HOTTIP (HoxA transcript at the distal tip) recruits MLL (Mixed lineage) via WDR5 (WD repeat-containing protein 5) leukemia) to the 5′ region of the HoxA gene cluster, which catalyzes the establishment of H3K4me3 and activates the expression of genes such as Hoxa11 and Hoxa13 in cis; Mira can form a DNA/RNA heterodimer with its locus and recruit MLL1, a member of the TrxG complex, which catalyzes the establishment of H3K4me3 and activates adjacent genes Hoxa6 and Hoxa7 expression, leading to differentiation of mES to the germline; Evf2 acts as a coactivator of DLX2 at high concentrations, enhancing Dlx5/6 enhancer activity and activating Dlx5 and Dlx6 transcription, while at low concentrations it can *cis*-suppress Dlx6 transcription through its Dlx6 antisense property, by recruiting MECP2, and thus HDAC Dlx5; asOct4-pg5 can recruit histone methyltransferases such as EZH2 and G9a to bind to the promoter regions of Oct4 and Oct4-pg5, establishing repressive chromatin modifications such as H3K27me3 and H3K9me3, which in turn repress transcription of Oct4 and Oct4-pg5, and when as Oct4-pg5 is combined with PURA (purine rich element binding protein A) and NCL (nucleolin), the ability to recruit EZH2 and G9a is lost and the repressive function is lost; the nascent ANRIL (antisense noncoding RNA in the INK4 locus) binds to CBX7 and ANRIL binds to CBX7 and promotes heterochromatin formation, while the formed ANRIL-CBX7 complex unbinds CBX7 to H3K27me3, leaving transcriptional repression in a dynamic state of flux. ANRIL recruits PRC2, allowing the INK4β/INK4α/ARF gene cluster to establish repressive modifications such as H3K27me3. ANRIL binds to SUZ12 and *cis*-represses INK4β transcription; DBE-T *cis*-recruits Ash1L to the 4q35 region, catalyzing the establishment of activating chromatin modifications such as H3K36me2, which activates gene transcription in the 4q35 region, ultimately leading to FSHD (facioscapulohumeral muscular dystrophy) disease (Guttman et al., 2011; Kopp and Mendell, 2018; Riaz and Li, 2019).

#### 1.2.3 LncRNAs Are Involved in Transcriptional Regulation

Xite and DXPas34 regulate Tsix expression in cis with enhancer activity; transcription of SER3 gene biosynthesis-associated 3-phosphoglycerate dehydrogenase in the biosynthesis of serine is regulated by lncRNA SRG1 (SER3 regulatory gene 1); Pwr1 interferes with the transcription of Icr1; Icr1 interferes with the transcription of Fol11; DHFRtinc interferes with the transcription of DHFR; Airn interferes with the transcription of Igf2r; Gas5 inhibits the binding of activated GR to target genes (Zhang et al., 2014; Ma et al., 2016b).

#### 1.2.4 LncRNAs Are Involved in Post-transcriptional Regulation

Malat1 regulates variable shear of Cat1 pre-mRNA; Zeb2-anti is involved in variable shear of Zed2; PTENpg1 asRNA β promotes PTENpg1 exit from the nucleus; Neat1 promotes retention of mRNA with IRAlus structure in the 3′ UTR region within paraspeckles; BACE1-AS increases BACE1 stability; MDRL as ceRNA promotes pri-miR484 maturation (Bian et al., 2019; He et al., 2020).

It has been reported that lncRNAs are usually involved in the progression of human-related diseases by such ways as being deregulated (Guttman et al., 2011; He et al., 2020), and some studies have shown that lncRNAs are involved in the key process of liver fibrosis by acting as regulators of HSC activation (Wang et al., 2014; Bian et al., 2019). Even though we can observe an impressive amount of literature suggesting an important role of lncRNAs in the liver fibrosis process, it is undeniable that the detailed mechanisms of lncRNAs in liver fibrosis remain unclear until now. In this review, we aim to provide a review of the latest developments in lncRNAs research, elaborate on the interactions between lncRNAs and miRNAs, and further evaluate the potential of lncRNAs as new therapeutic targets in liver fibrosis.

## 2 The Regulatory Role of lncRNAs in Liver Fibrosis

The distribution of lncRNAs in liver fibrosis has been detected by the latest high-throughput methods such as next-generation sequencing and microarrays (Zheng et al., 2015; Xiong et al., 2016), and the pleiotropic nature of lncRNAs has been demonstrated in the activation and apoptosis of HSCs and the progression of multiple liver fibrosis by interacting with molecules such as miRNAs, specific structural domains, and proteins to regulate key genes in liver fibrosis, thus exerting their potential (Bu et al., 2020). In this paper, we review the role of lncRNAs in liver fibrosis and their potential mechanisms in the development of liver fibrosis. Table 1 provides a summary of the expression patterns, functional roles, and regulatory mechanisms of lncRNAs.

**TABLE 1:** The expression of lncRNA in liver fibrosis.

lncRNAs	Expression	Role	Functional role	References
lncRNA NEAT1	Upregulated	Promotion of liver fibrosis	HSC activation, inflammatory response	Yu et al. (2017b); Ye et al. (2020)
lncRNA SNHG7	Upregulated	Promotion of liver fibrosis	HSC activation, autophagy and proliferation, survival, cell cycle	Yu et al. (2019); Xie et al. (2021)
lncRNA H19	Upregulated	Promotion of liver fibrosis	proliferation, activation, metabolism of lipid droplets, *trans*-differentiation	Song et al. (2017); Wang et al. (2020a)
lncRNA MALAT1	Upregulated	Promotion of liver fibrosis	HSC proliferation, cell cycle, and activation	Yu et al. (2015a); Wu et al. (2015)
lncRNA HOTTIP	Upregulated	Promotion of liver fibrosis	HSC cell proliferation and activation	Li et al. (2018a); Zheng et al. (2019)
lncRNA TUG1	Upregulated	Promotion of liver fibrosis	HSC activation	Han et al. (2018); Zhang et al. (2020a)
lncRNA HULC	Downregulated	Inhibition of liver fibrosis	Hepatic steatosis, inflammation, hepatocyte red lipid vesicles, HSC apoptosis	Shen et al. (2019a)
lincRNA-p21	Downregulated	Inhibition of liver fibrosis	HSC activation, proliferation, apoptosis	Yu et al. (2017c); Tu et al. (2017)
lncRNA MEG3	Downregulated	Inhibition of liver fibrosis	HSC activation, proliferation, EMT	Yu et al. (2018); Chen et al. (2019)
lncRNA GAS5	Downregulated	Inhibition of liver fibrosis	HSC activation, EMT	Yu et al. (2015b); Dong et al. (2019a)

### 2.1 LncRNAs Involved in the Promotion of Liver Fibrosis

#### 2.1.1 LncRNA HULC

Panzitt et al. identified for the first time the highly up-regulated hepatocellular carcinoma LncRNA (HULC) of approximately 500 nucleotides containing two exons located on chromosome 6p24.3 as the most highly expressed lncRNA in human hepatocellular carcinoma, whose transcribed RNA does not have a considerable open reading frame nor does it produce any protein (Panzitt et al., 2007). The HULC promoter and its first exon are in a long terminal repeat sequence (LTR) retrotransposon-like sequence (Kapusta et al., 2013). The upregulation trend of HULC can be observed in all accessible studies on hepatocellular carcinoma (HCC) (Chen et al., 2017; Xin et al., 2018; Ghafouri-Fard et al., 2020b). Several literatures have reported that HULC is upregulated in cancer and is regulated as an oncogene lncRNA in tumorigenesis and progression (Kitagawa et al., 2013; Parasramka et al., 2016). Considering its high expression in HCC cells, previous studies have also shown the potential of HULC as a novel antitumor therapeutic agent (Klec et al., 2019). cAMP response element binding protein (CREB) is usually bound to and activated by the target promoter (Mayr and Montminy, 2001; Kong et al., 2016), and Wang et al. demonstrated the presence of CREB binding sites in the HULC promoter region and the ability to further activate the HULC promoter (Wang et al., 2010), which can affect the expression of HULC at the transcriptional level (Shen X. et al., 2019). Shen et al. revealed the role of lncRNA HULC in the progression of liver fibrosis in rats with nonalcoholic fatty liver disease (NFALD) and that inhibition of HULC suppressed steatosis. The degree of hepatic steatosis, inflammation, hepatocyte red lipid vesicles and apoptosis were also significantly reduced with the knockdown of HULC gene. Inhibition of HULC significantly reduced liver fibrosis scores and liver fibrosis indices (HA, LN, PC III, and IV-C) (Shen X. et al., 2019). Inhibition of HULC improved liver fibrosis and reduced hepatocyte apoptosis in NAFLD rats. In general, the above findings not only provide valuable candidate molecular markers for liver fibrosis and indicators of advanced liver fibrosis but also provide new insights into the role of lncRNA in the biology of cancer.

#### 2.1.2 LncRNA Nuclear Paraspeckle Assembly Transcript 1

Nuclear paraspeckle assembly transcript 1 (NEAT1) was characterized as an unusual RNA polymerase II transcript that lacks introns and accompanied by non-canonical processing of the non-polyadenylated 3′-end by RNase P (Ding et al., 2019). NEAT1 was found to be upregulated in gastric adenocarcinoma and human laryngeal squamous cell carcinoma (Ma et al., 2016a; Wang et al., 2016), which suggested that it promotes tumor development by promoting cell proliferation and survival as well as inhibiting apoptosis (Choudhry et al., 2015). A similar situation has been observed in hepatocellular carcinoma (Guo et al., 2015). Yu et al. examined NEAT1 expression in cCl4-induced mice. qRT-PCR analysis showed increased expression of NEAT1 in CCl4-treated livers compared to control livers, and a significant increase in NEAT1 expression in HSCs was also observed during different weeks of CCl4 treatment (Yu et al., 2017b), Huang et al. screened the aberrantly expressed microRNAs in the CCl4-induced mouse liver fibrosis model by analyzing the GSE77271 microRNA microarray based on the Agilent-046065 mouse miRNA V19.0 platform. Neat1 simultaneously targeted miR-148a-3p and miR-22-3p, and showed the most significant increase in liver fibrosis mice that displayed the most marked increase in expression upregulation, and its expression in the CCl4 group exceeded 2-fold that of the control group (Huang et al., 2021), and inhibition of NEAT1 was observed to reverse isotropic liver fibrosis with concomitant reduction in α-SMA and type I collagen content, which was further confirmed by NEAT1 knockdown assays and NEAT1 overexpression assays (Yu et al., 2017b). A similar situation was confirmed in the alcoholic steatohepatitis (ASH) assay by Ye et al. (Ye et al., 2020). Inhibition of NEAT1 suppressed ethanol-stimulated elevated lipid metabolism and inhibited inflammatory responses in AML-12 cells. More importantly, inhibition of NEAT1 upregulated ethanol-induced hepatic function in ASH mice and inhibited lipid, inflammatory responses, hepatocyte apoptosis, and hepatic fibrosis, demonstrating that knockdown of NEAT1 inhibited hepatic fibrosis in ASH mice and thus slowed down the development of ASH (Ye et al., 2020). Related mechanistic studies suggest that Kruppel-like factor 6 (KLF6), as an important pro-fibrotic gene, is involved in the regulation of liver fibrosis by NEAT1 (Yu et al., 2017b), and that NEAT1 overexpression induces KLF6 mRNA and protein expression. However, it is of interest that KLF6 knockdown experiments showed NEAT1-induced proliferation of HSC, while KLF6 siRNA blocked NEAT1-induced α- SMA and type I collagen production, suggesting that NEAT1 could mediate HSC activation through KLF6 (Yu et al., 2017b). Huang et al. suggested that NEAT1 knockdown could inhibit the process of liver fibrosis and HSCs activation by regulating the expression of a cellular adhesion element 3 (Cyth3) associated with allosteric insulin signaling in mammals (Jux et al., 2019; Huang et al., 2021). And Ye et al. further identified that downregulation of NEAT1 could limit the inflammatory response and liver fibrosis in ASH mice by reducing suppressor of cytokine signaling 2 (SOCS2) (Ye et al., 2020), which is a feedback inhibitor of the growth hormone/insulin-like growth factor axis (Monti-Rocha et al., 2018).

#### 2.1.3 LncRNA Small Nucleolar RNA Host Gene 7

It was first reported by Chaudhry in 2013 that a new full-length 2,176 bp oncogenic lncRNA, known as lncRNA small nucleolar rna host gene 7 (SNHG7), expressed in lymphoblastoid cell lines TK6 and WTK1 (Chaudhry, 2013), which is located on chromosome 9q34.3. Recent studies have shown a significant increase in its expression in tumor cells of digestive system, breast, and prostate (Wu F. et al., 2020; Wu X. et al., 2020), and further studies have demonstrated that SNHG7 is widely involved in the proliferation, invasion and migration of various tumor cells (Xia et al., 2020), including its regulation in the progression of HCC and liver fibrosis (Cui et al., 2017). Just as Xie et al. found increased expression of SNHG7 in primary HSC mice as well liver fibrosis, suggesting its regulation of HSC activation (Xie et al., 2021), and SNHG7 knockdown experiments showed decreased expression levels of α-SMA and Col. I (Xie et al., 2021), similarly SNHG7 inhibition was associated with reduced survival and proliferation rates in liver fibrosis mice. Current studies have identified several types of high confidence indicators of autophagy, such as the cytoplasmic form of LC3, a key protein in autophagosome formation (LC3-I), the active membrane-bound form of LC3 (LC3-II), and Beclin1 (Wirawan et al., 2012; Alirezaei et al., 2015; Dodson et al., 2017; Feng et al., 2017). Xie et al. revealed that knockdown of SNHG7 could reduce the decrease the expression level of Beclin1, LC3-II and LC3-I ratio, demonstrating the inhibitory effect of SNHG7 knockdown on HSC autophagy (Xie et al., 2021). DNMT3A induces a DNMT-regulated DNA ab initio methylation process, and DNA methylation/hydroxy methylation, a key step in HSC activation and liver fibrosis development, can be inhibited by activation of DNMT3A expression in HSCs (Garzon et al., 2009; Page et al., 2016). Several recent mechanistic studies suggest that SNHG7 knockdown is significantly associated with low expression levels of DNMT3A. These results confirm the relationship between SNHG7 and DNMT3A, which are novel regulators of HSC activation, autophagy, and proliferation in liver fibrosis (Xie et al., 2021). Yu et al. identified a positive correlation between SNHG7 levels and type I collagen mRNA levels in patients with cirrhosis (Yu et al., 2017b). In addition, SNHG7 showed a significant association in regulating activated HSCs proliferation and the cell cycle associated with increased G0/G1 phase cells and decreased S phase cells. SNHG7 knockdown experiments performed in activated HSCs inhibited type I collagen expression (Yu et al., 2017b), as well as collagen deposition and hydroxyproline due to carbon tetrachloride were similarly blocked by silencing of SNHG7 *in vivo*, suggesting that inhibition of liver fibrosis can be mediated by downregulation of SNHG7 (Yu et al., 2017b). Furthermore, Yu et al. demonstrated at the mechanistic level the role of SNHG7 in regulating the expression level of irregular fragment polarity protein 2 (DVL2) (Yu et al., 2017b; Nielsen et al., 2019), which was positively correlated with DVL2, the deletion of which also blocked its effect on HSCs activation (Yu et al., 2017b). In conclusion, all these data suggest that SNHG7 is an impressive possible therapeutic target and a potential diagnostic marker for liver fibrosis.

#### 2.1.4 LncRNA H19

LncRNA H19 is expressed only by the maternal allele 11p15.5, which can encode 2.3 kb RNA and is transcribed by RNA polymerase II (Gabory et al., 2006), splicing and polyadenylation (Ghafouri-Fard et al., 2020a). It is exported from the nucleus to the cytoplasm, adjacent to the insulin-like growth factor 2 (IGF2) gene, and they are expressed from the maternal and paternal genetic chromosomes, respectively (Raveh et al., 2015; Wang J. et al., 2020). H19 RNA molecules have now been observed to be present in the cytoplasm at much higher levels than in the nucleus. H19 plays an essential role in biological processes such as apoptosis, angiogenesis, inflammation and cell death through regulatory RNA or ribosomal regulators (Yoshimura et al., 2018). This includes the regulation of proliferation, invasion, and metastasis processes in a variety of tumors of the digestive system (Zhou et al., 2017; Wei LQ. et al., 2019). Multiple complex mechanisms have been demonstrated in different cancers (Zhang D. M. et al., 2017; Zhou et al., 2017). Of interest is the upregulation of the level of intracellular transcripts (Zhu et al., 2019) and extracellular exosomes (Li X. et al., 2018), known as lncRNA-H19, observed in activated HSCs, which is thought to be associated with HSCs-activated metabolic processes like lipid (Liu et al., 2018) and cholesterol metabolism (Xiao et al., 2019). Previous research concluded that the level of fibrosis in the liver was positively correlated with the level of H19, and that H19 knockdown attenuated Bcl-2-induced liver injury (Zhang Y. et al., 2016), while conversely H19 overexpression significantly exacerbated the process of HSCs and EMT activation in hepatocytes (Zhu et al., 2019). Song et al. demonstrated the overexpression of H19 in bile duct ligation (BDL)-induced liver fibrosis with abnormal liver function parameters (Song et al., 2017), and identified a new downstream target gene of ZEB1, called EpCAM (Song et al., 2017), which promotes cholestatic liver fibrosis by interacting with the ZEB1 protein to prevent its binding to the EpCAM promoter and thus the inhibitory effect of ZEB1 (Song et al., 2017). Liu et al. reported that cholangiocyte-derived exosomal H19 promotes cholestatic liver injury in Mdr2^−/−^ mice and promotes HSCs transdifferentiation and activation, along with upregulation of fibrotic gene expression in HSCs-derived fibroblasts (Liu et al., 2019). Wang et al. discovered that H19 can promote RARα and RXRβ mRNA and protein synthesis (Wang ZM. et al., 2020), and its reduced expression reversed the extent of HSCs activation induced due to increased retinoic acid signaling. Meanwhile, it should be mentioned that H19 knockdown-mediated HSCs inactivation was inhibited by the activation of retinoic acid signaling. Furthermore, they demonstrated that H19 enhancement was positively associated with a synergistic increase in retinoic acid metabolism during HSCs activation (Wang ZM. et al., 2020). More significantly, they confirmed that inhibition of ethanol dehydrogenase III (ADH3) completely abolished the effect of H19-mediated retinoic acid signaling, and that dihydroartemisinin (DHA), a natural inhibitor of H19, reduced both H19 and ADH3 expression and thus inhibited HSC activation (Wang ZM. et al., 2020). Taken together, these results reveal some of the molecular mechanisms underlying the increase in retinoic acid signaling during HSCs activation and suggest that the lncRNA-H19/ADH3 pathway is a potential target for the treatment of liver fibrosis. Similarly, H19 expression levels were increased in CCl_4_-induced fibrotic liver thereby activating HSCs (Wang Z. et al., 2020). Further studies revealed the role of hypoxia-inducible factor-1α (HIF-1α) in promoting H19 expression by binding to the H19 promoter at two hypoxia response element (HRE) sites located at 492–499 and 515–522 bp (Wang Z. et al., 2020). H19 knockdown experiments also resulted in significant inhibition of HSC activation and attenuated liver fibrosis, suggesting that lncRNAH19 may be a potential target for antifibrotic therapeutic approaches. Moreover, the H19 silencing assay reduced the degree of lipid oxidation and the H19 knockdown assay restored the levels of lipid droplets, triglycerides, cholesteryl esters and retinyl esters in HSCs without changes in lipid uptake and synthesis (Wang Z. et al., 2020). In conclusion, as described above, the results highlight the role of H19 in the proliferation, activation and metabolism of lipid droplets in HSCs and reveal its feasibility as a new molecular target to attenuate liver fibrosis.

#### 2.1.5 LncRNA Metastasis-Associated Lung Adenocarcinoma Transcript1

Ji et al. characterized a metastasis-associated lung adenocarcinoma transcript (MALAT1) transcribed by RNA polymerase II located on human chromosome 11q13 and mouse chromosome 19qA (Zhang et al., 2012; Wilusz, 2016), which is widely known for its properties in predicting early NSCLC metastasis and survival (Ji et al., 2003), the major transcript of MALAT1 is approximately mid-8 kb in humans and 6.7 kb in mice (Wilusz et al., 2008). MALAT1-associated small cytoplasmic RNA (mascRNA) is a larger fragment of approximately 6.7 kb and a smaller fragment of 61 nucleotides produced by the action of ribonuclease P and ribonuclease Z on MALAT1 (Brown et al., 2014), while the larger fragment or mature transcript is highly stable due to a unique triple-helix structure at the 3′ end that protects it from nucleic acid exonucleases (Zhang et al., 2012). The highly conserved and widespread expression of MALAT1 in mammalian tissues and cancers implies its functional importance; MALAT-1 dysregulation in a variety of cancers has been extensively studied. In most cases, it functions as a promoting role in the development of different types of tumors (Kim et al., 2018; Feng et al., 2019). MALAT1 upregulation is closely associated with the development of cancers such as lung (Wei S. et al., 2019), glioblastoma (Voce et al., 2019), esophageal squamous cell carcinoma (Chen M. et al., 2018), renal cell carcinoma (Zhang H. et al., 2019), colorectal cancer (Zhang H. et al., 2019; Xie et al., 2019), osteosarcoma (Chen Y. et al., 2018), multiple myeloma (Amodio et al., 2018), gastric cancer (Zhang YF. et al., 2020), gallbladder cancer (Lin et al., 2019), and other cancers (Tian and Xu, 2015), as well as other clinicopathological features including tumor location, tumor size, differentiation and tumor stage (Goyal et al., 2021). Numerous studies have shown that as a biomarker for tumor diagnosis and prognosis, the abnormal expression of MALAT1 in tumor tissues and/or body fluids is highly indicative (Leti et al., 2017; Peng et al., 2018). In a physical and functional interaction study with the liver fibrosis process, Yu et al. found that MALAT1 expression was significantly upregulated in fibrotic liver tissues and simultaneously activated HSCs (Yu et al., 2015a), while silencing MALAT1 suppressed the mRNA levels of α-SMA and Col.I and downregulated the protein levels of α-SMA and collagen type I in HSC respectively (Yu et al., 2015a). Sirius red staining of collagen in mouse liver tissue resulted in the observation that mice transduced by silencing MALAT1 showed a 54% downregulation of collagen accumulation compared to CCl4-treated mice (Yu et al., 2015a), reflecting the role of MALAT1 in accelerating the progression of liver fibrosis *in vivo*. Dai et al. used arsenite treatment of L-02 cells as well as co-culture of LX-2 cells and found that MALAT1 expression levels increased as well as co-culture promoting activation of LX-2 cells (Dai et al., 2019). They further discovered that MALAT1 levels were increased in exosomes of arsenite-treated L-02 cells and LX-2 cells exposed to exosomes from arsenite-treated L-02 cells (Dai et al., 2019), and these exosomes also promoted LX-2 cell activation; blocking MALAT1 expression simultaneously inhibited these changes, thus suggesting a mechanism by which MALAT1 induces LX-2 cell activation via exosomes. Silent information regulator 1 (SIRT1), as a member of the mammalian sirtuin family of proteins (SIRT1-SIRT7) (Dai et al., 2019), is homologous to the yeast Sir2 protein, and SIRT1 is involved in a variety of biological processes and exhibits multiple physiological functions through the deacetylation of many non-histone proteins (Houtkooper et al., 2012). Wu et al. verified the important role of SIRT1 in hepatic stellate cell activation and reversal and its overexpression counteracting TGF-β1-induced LX-2 cell activation (Wu et al., 2015), suggesting its potential as an alternative for the treatment of liver fibrosis. Further studies found that the evolutionarily highly conserved MALAT1 has a strong tendency to interact with SIRT1 (discriminatory power 100%) (Wu et al., 2015), which was verified in CCL4-treated mice and LX-2 cells exposed to TGF-β1, considering that the expression level of MALAT1 mRNA was significantly upregulated and accompanied by negative changes in SIRT1 protein (Wu et al., 2015). MALAT1 silencing assay yielded results that eliminated the TGF-β1-induced upregulation of myofibroblast markers and the downregulation of SIRT1 protein. These phenomena suggest a role for MALAT1 in mediating the expression as well as the function of SIRT1 in regulating liver fibrosis. In conclusion, these findings highlight the role of MALAT1 in liver fibrosis and suggest a mechanism for fibrosis development (Wu et al., 2015). However, future efforts should be devoted to elucidating other regulatory mechanisms and clinical implications of MALAT1 in liver fibrosis.

#### 2.1.6 LncRNA HOXA Transcript at the Distal Tip

HOXA transcript at the distal tip (HOTTIP) is a functionally characterized lncRNA (Li et al., 2016). Wang et al. demonstrated that the HOTTIP gene is located at chromosomal locus 7p15.2 and encodes a 4665 bp transcript (Wang et al., 2011). Its function is to directly interact with the Trithorax protein WDR5 and induce open DNA chromatin conformation, target the WDR5/MLL complex and drive histone H3 lysine 4 trimethylation for transcriptional regulation of the 50-terminal HOXA locus gene (Wang et al., 2011). This suggests that HOTTIP is not only involved in developmental processes but also enhances the effect of this lncRNA as a cancer-associated lncRNA considering its role as a signaling transmitter from higher-order chromosome conformation to chromatin coding (Wang et al., 2011). Overall survival (OS), distant metastasis (DM), lymph node metastasis (LNM), and tumor staging of human tumors have been extensively studied and determined to be closely associated with HOTTIP expression, suggesting that HOTTIP expression may influence the prognosis and metastasis of several human cancers (Broerse and Crassini, 1984; Quagliata et al., 2014). The most representative case is the high HOTTIP expression in human HCC specimens (Quagliata et al., 2014) and its close correlation with clinical progression and disease outcome (Tsang et al., 2015). It is worth mentioning that HOTTIP has long been shown to be dysregulated in the early stages of hepatocellular carcinoma formation, and recent studies suggest a positive correlation between its expression and liver fibrosis progression (Yang et al., 2019; He et al., 2020). Zheng et al. verified the specific expression status of HOTTIP in liver fibrotic tissue and primary quiescent HSC (Zheng et al., 2019), and their qRT-PCR results showed a 22.6-fold increase in HOTTIP expression on day 10 compared to day 2 increased 22.6-fold, similar to the results from the group of oil-treated mice compared to the group of CCl_4_-treated mice suggesting a significant upregulation of HOTTIP expression in HSCs however this phenomenon was not observed in hepatocytes (Zheng et al., 2019). Furthermore, the mRNA and protein levels of α-SMA and Col. I was also found to be reduced by hot-end silencing but Edu incorporation assay demonstrated that hot-end downregulation inhibited the proliferation of activated HSCs. The above results suggest that HOTTIP downregulation could inhibit HSCs activation and proliferation. Related mechanistic studies suggest that HOTTIP is a target of miR-150 and is also recruited to Ago2-associated miRNPs (Zheng et al., 2019), possibly acting through miR-150 association. In addition, bioinformatics analysis and luciferase analysis of a series of experiments also confirmed the role of serum response factor (SRF) as a target of miR-150. They further demonstrated that the inhibition of HSCs activation was caused by an increase in SRF mRNA expression due to HOTTIP overexpression (Zheng et al., 2019). Li et al. revealed that HOTTIP expression was significantly upregulated in fibrotic and cirrhotic liver samples, with the highest in cirrhotic samples (Li Z. et al., 2018), and this was also found in liver fibrotic tissue, primary HSC and activated LX-2 cells. Inhibition of HOTTIP at the mRNA and protein levels was effective in reducing the expression of α- SMA and Col. I. They found that downregulation of HOTTIP attenuated CCl_4_-induced liver fibrosis in mice. In contrast, the relative survival of HSC in LX-2 cells and the mRNA and protein levels of α-SMA and Col. I were significantly reduced by HOTTIP knockdown (Li Z. et al., 2018). Li et al. proposed a possible mechanism to promote HSC activation, i.e., negative regulation of HOTTIP mediated by miR-148a, considering that TGFBR1 and TGFBR2 were identified as miR-148a novel targets in HSCs. TGFBR1 and TGFBR2 levels were increased by high levels of HOTTIP, which led to the progression of liver fibrosis (Li Z. et al., 2018). These results highlight the potential of the HOTTIP/miR-148a/TGFBR1/TGFBR2 axis as a potential marker and target in patients with liver fibrosis. In conclusion, HOTTIP promotes HSCs cell proliferation and activation suggesting its possible role as a fibrogenic gene in liver fibrosis and plays a key role as a prognostic marker and novel therapeutic target. However, these still need to be investigated further.

#### 2.1.7 LncRNA Taurine Upregulated Gene 1

The 7,598 nucleotide lncRNA sequence localized on chromosome 22q12.2, also known as taurine upregulated gene 1 (TUG1), was initially identified in a genomic screen of taurine-treated mouse retinal cells (Young et al., 2005; Zhang et al., 2013). Functional studies in mice further demonstrated that knockdown of TUG1 inhibits retinal developmental processes (Khalil et al., 2009). Khalil et al. demonstrated by whole genome RNA immunoprecipitation analysis that approximately 20% of lncRNAs (including TUG1) with methyltransferase activity promote demethylation and trimethylation of lysine residue 27 of histone 3 (H3K27me3) in the target gene and inhibit its expression by binding to polyclonal repressor complex 2 (PRC2), which inhibits its expression (van Kruijsbergen et al., 2015). Besides other PRC2-associated lncRNAs involved in tumorigenesis and progression, TUG1 regulates the biological behavior and molecular mechanisms of different cancer cells, including cell proliferation, invasion, apoptosis, differentiation, migration, drug resistance, radiation resistance, angiogenesis, mitochondrial bioenergetics, epithelial-mesenchymal transition (EMT), and regulation of blood-tumor barrier permeability among other different cancer cell (Niland et al., 2012; Katsushima et al., 2016; Cai et al., 2017; Chiu et al., 2018). TUG1 is closely associated with the mediation of radio resistance and angiogenesis in hepatoblastoma (Dong et al., 2016). TUG1 has also been extensively studied in liver diseases such as cirrhosis and liver fibrosis. Zhang et al. demonstrated that TUG1 is highly expressed in liver sinusoidal endothelial cells (LSEC), and the results of TUG1 knockdown experiments revealed inhibition of the extent of expression of autophagy and EMT-related genes (Zhang R. et al., 2020). In contrast, knockdown of TUG1 eliminated the most significant increase in autophagy-related genes in LPS-treated LSEC under starvation. The increase in ATG5 expression while inhibition of ATG5 attenuated autophagy and EMT (Zhang R. et al., 2020). Han et al. demonstrated that TUG1 was overexpressed in liver samples from patients with CCl4 and BDL-induced liver fibrosis *in vivo* as well as cirrhosis and activated HSCs while promoting a degree of expression of SMA, Col1a1, Mmp2/9/10, and Timp1. The possibility that TUG1 accelerates the progression of liver fibrosis by promoting the expression of these pro-fibrotic genes through downregulation of miR-29b is mechanistically argued (Han et al., 2018). Collectively, these studies revealed the mechanisms of TUG1 play a crucial role in liver fibrosis, suggesting its ability to monitor human liver fibrosis and its potential to be a candidate biomarker for new therapeutic strategies.

### 2.2 LncRNAs Involved in the Inhibition of Liver Fibrosis

#### 2.2.1 Long Intergenic Non-Coding RNA p21

LincRNA-p21 (long intergenic non-coding RNA p21) localized at human chromosome 6p21.2 situated approximately 15 kb upstream of the cell cycle regulatory gene p21/Cdkn1a and approximately 3.0 kb in length has been described as an inducer of p53-dependent apoptosis in mouse embryonic fibroblasts (Huarte et al., 2010). lincRNA-p21 is available in two types both containing an exon and an Alu isoforms with a reverse repeat element (Yoon et al., 2012a; Yoon et al., 2012b; Wilusz and Wilusz, 2012). Coordinates the degree of autoregulation and expression of its target transcripts by interacting with RNA-binding proteins, miRNA, and mRNA targets (Yoon et al., 2012a). As a transcriptional target of p53 it is involved in the p53 pathway, downregulating many p53 target genes and triggering the apoptotic process by physically interacting with the p53 repressor complex (Fatica and Bozzoni, 2014). lincRNA-p21 has also been reported to regulate gene expression by directing protein binding partners in chromatin localization and thus directly binding to target mRNAs to act as a translational repressor and thus by activating p21 in cis participate in the regulation of the G1/S checkpoint (Dimitrova et al., 2014). It is also noteworthy that it can feedback regulate p53 activity by regulating the interaction of p53, p300, and MDM2 (Wu et al., 2014; Tang et al., 2015), thus participating in different tumorigenesis including hepatocellular carcinoma (Jia et al., 2016). In terms of tumor invasion lincRNA-p21 overexpression can be inhibited by Notch pathway (Wang et al., 2017). Besides it plays a key regulatory role in DNA damage response, apoptosis, and cell proliferation among other different processes (Ozgur et al., 2013). Zheng et al. observed in animal experiments that lincRNA-p21 expression was downregulated in liver fibrosis (Zheng et al., 2015). lincRNA-p21 was negatively correlated with disease progression and HSCs activation status, while *in vitro* and *in vivo* distribution inhibited HSCs activation and reduced liver fibrosis progression. Notably the reversibility of the inhibitory effect of lincRNA-p21 was confirmed by the removal of lincRNA-p21 leading to classical morphological changes associated with HSCs activation. lincRNA-p21 was found by Zhang et al. to inhibit the cell cycle and proliferation of primary HSCs by enhancing p21 (Zheng et al., 2015), while Tu et al. found a significant increase in hepatocyte lincRNA-p21 expression during hepatic fibrosis (Tu et al., 2017). These suggest that lincRNA-p21 contributes to a positive role in hepatocyte apoptosis and inhibition of hepatocyte growth in fibrotic livers. Knockdown of hepatocyte lincRNA-p21 attenuated CCl_4_-induced hepatocyte apoptosis thereby reducing CCl_4_-induced inflammatory cell infiltration and secretion levels of pro-inflammatory and pro-fibrotic cytokines in the fibrotic liver. Mechanistic studies have shown that inhibition of miR-30 impairs the effect of lincRNA-p21 in the development of liver fibrosis (Tu et al., 2017). lincRNA-p21/miR-30 axis has been highlighted as a potential marker and target for patients with liver fibrosis. Yang et al. found that lincRNA-p21 overexpression promotes hepatocyte apoptosis, but its results can be blocked by thymosin β4 (Tβ4) blocked, and additionally Tβ4 reversed lincRNA-p21- induced cleavage of caspase-3 and caspase-9 levels (Tu et al., 2017). LincRNA-p21 overexpression increases the levels of fibrosis-associated proteins (type I collagen, α- SMA, and TIMP-1) and induces hydroxyproline and ALT production leading to pathological damage of liver tissue and progression of fibrosis. The potential utility of lincRNA-p21 in predicting cirrhosis is supported by the results of downregulation of serum lincRNA-p21 levels in cirrhotic patients (Yang L. et al., 2020). Yu et al. reported a decrease in serum lincRNA-p21 levels in patients with chronic hepatitis B that negatively correlated with the stage of liver fibrosis, thus revealing its diagnostic value (Yu et al., 2017c). There was also a negative correlation between serum lincRNA-p21 levels and markers of liver fibrosis (including α-SMA and Col. I) but not in markers of viral replication, liver inflammatory activity and liver function. The primary HSC culture results suggested that the deletion of lincRNA-p21 expression was associated with promoter methylation, and these conditions implied the potential of serum lincRNA-p21 as a potential biomarker of liver fibrosis in patients with chronic hepatitis B/cirrhosis. Promoter methylation may be involved in the downregulation of lincRNA-p21 in liver fibrosis (Yu et al., 2017c). Collectively, these findings demonstrate the ability of lincRNA-p21 to act as a mediator of HSCs activation and proliferation, suggesting its potential as a new therapeutic target for liver fibrosis.

#### 2.2.2 LncRNA Maternally Expressed Gene 3

The maternally expressed gene 3 (MEG3), located within the human chromosome 14q32.3 DLK1-MEG3 locus (Wylie et al., 2000), is 35 kb in size consisting of 10 exons (Zhou et al., 2012) and encodes an approximately 1.6 kb long non-coding RNA as a contained 10 exons (Zhang et al., 2010). Selectively spliced transcripts of Gtl2 (gene trap site 2 (Gtl2) is the mouse homolog of human MEG3) extend to contain intron-encoded C/D box SNORNAs and miRNAs, suggesting that Gtl2 may function as a host gene for these small RNAs (Cavaille et al., 2002; Lin et al., 2003; Tierling et al., 2006). MEG3 can be observed in unimprinted embryonic cells to silence genes involved in neurogenesis by regulating the chromatin targeting of multicomb proteins and plays an important role in neuronal development (Mercer et al., 2008; Kaneko et al., 2014; Mondal et al., 2015). Recent studies have suggested that MEG3 may act as a tumor suppressor considering the extent to which its loss of expression in several cancers is associated with inhibition of cell proliferation (Ghafouri-Fard and Taheri, 2019). Yu et al. showed that the process of liver fibrosis is accompanied by a decrease in MEG3 *in vivo* and *in vitro* and that restoration of MEG3 expression inhibits liver fibrosis while reducing α-SMA and type I collagen production (Yu et al., 2018). MEG3 overexpression inhibits HSC activation through EMT and is associated with E-calcium activation. The Hedgehog (Hh) pathway is one of the pathways involved in HSC activation by MEG3 as an EMT process. Smoothing (SMO) plays an important role in the Hh pathway. Bioinformatics analysis, RNA immunoprecipitation and deletion mapping results suggest that the interaction between MEG3 and SMO is involved in EMT repression caused by MEG3 overexpression (Yu et al., 2018). Gene expression in the DLK1-MEG3 region is controlled by two differentially methylated regions (DMRs) consisting of multiple methylated CpG sites located approximately 13 kb upstream of the MEG3 transcription start site intergenic DMR (IG-DMR) and overlaps with a 1.5-kb upstream promoter in the post-fertilization-derived secondary (MEG3-DMR) (Murphy et al., 2003), indicating the important role that DNA methylation plays in silencing the MGE3 gene (Anwar et al., 2012). The most widely studied epigenetic modification, DNA methylation and its relevance to the pathogenesis of liver fibrosis have been well established experimentally (Benetatos et al., 2008; Li et al., 2010), and previous studies have suggested a role for DNA methylation in the deletion of MEG3 expression in tumors (Zhao et al., 2005; Benetatos et al., 2008). He et al. revealed that MEG3 levels were significantly reduced in CCl4-induced liver fibrosis in mice and humans, while MSP was significantly reduced in CCl4-treated mouse liver tissue and human liver fibrosis tissue and TGF-β1-treated LX-2 cells where MEG3 promoter methylation was observed (He et al., 2014). The effect of 5-azadC to block MEG3 methylation could be achieved by the methylation inhibitor 5-azadC significantly eliminating TGF-β1-induced aberrant MEG3 hypermethylation and restoring MEG3 in TGF-β1-treated LX-2 cells thereby inhibiting HSC activation and proliferation expression illustration (He et al., 2014). The inhibition of activation and the degree of proliferation of LX-2 cells and the reversal of methylation of the MEG3 promoter were both closely associated with the deletion of DNMT1 thereby restoring MEG3 expression. While 5-azadC treatment or knockdown of DNMT1 downregulated mRNA and protein production of α-SMA and Col. I in TGF-β1-treated LX-2 cells, overexpression of MEG3 was detected in TGF-β1-treated LX-2 cells (He et al., 2014), which significantly activated p53 protein levels and induced a Bax/Bcl-2 ratio accompanied by a significant increase in cytoplasmic cytochrome c significantly increased. These suggest that the p53-dependent mitochondrial apoptotic pathway is partially involved in the MEG3-induced apoptosis process (He et al., 2014). In conclusion, these findings demonstrate that MEG3 may play an important role in stellate cell activation and liver fibrosis progression and presents as a new potential treatment target for liver fibrosis.

#### 2.2.3 LncRNA GAS5

Situated at 1q25 and composed of 12 exons, GAS5 was originally identified from a subtractive cDNA library, named according to the increased level of expression found in mammalian cells at growth arrest (Sun et al., 2017). Its exons are selectively spliced to produce two possible mature lncRNAs: GAS5a and GAS5b (Li J. et al., 2018) and 11 introns responsible for encoding 10 cassettes of C/D small nucleolar RNA (snoRNA) (Ni et al., 2019). Sequence similarity to the hormone receptor element of the glucocorticoid receptor (GR) in terms of function inhibits the effect of GR on its target gene expression (Zhong et al., 2020). Considering other regions of sequence similarity suggests a role for this lncRNA in regulating the function of other hormones such as androgen, progesterone, and salt corticosteroid receptors (Dong P. et al., 2019; Yang X. et al., 2020). Additionally, plasma GAS5 is involved during diabetes and coronary heart disease. Yu et al. showed that GAS5 could directly bind to miR-222 in mouse, rat and human fibrotic liver samples as well as in activated HSC but its overexpression inhibited the activation of primary HSC *in vitro* while attenuating collagen accumulation levels in fibrotic liver tissues *in vivo*, but this was not observed in response to GAS5 is predominantly localized in the cytoplasm (Yu et al., 2015b) accompanied by a higher copy number than miR-222 and is noted to increase p27 protein levels by binding to miR-222, thereby acting as a suppressor in HSC activation and proliferation (Yu et al., 2015b). Han et al. revealed that GAS5 expression was strongly correlated with liver fibrosis in patients with nonalcoholic fatty liver disease (NAFLD) (Han et al., 2020), and plasma GAS5 expression was significantly higher in patients with advanced stages than in non-advanced stages (Han et al., 2020). The progression of fibrosis was linearly correlated with plasma GAS5 expression, which also suggests the potential of plasma GAS5 as a noninvasive marker of liver fibrosis in patients with NAFLD (Dong Z. et al., 2019). Dong et al. investigated CCl4-induced *in vivo* assays in model rats and TGF-β1-induced *in vitro* assays in HSC and found that miR-23a expression was significantly increased while compared with miR-23a Compared with the NC group (Dong Z. et al., 2019), miR-23a inhibitor did not affect the expression levels of E-calmodulin, α-SMA and type I collagen in normal rats while up-regulating the expression levels of E-calmodulin and down-regulating the expression levels of α-SMA and type I collagen in model rats, suggesting that miR-23a plays a critical regulatory role in the development of liver fibrosis (Dong Z. et al., 2019). Further co-transfection revealed that the relative luciferase activity of pGL3-GAS5-wt was inhibited by miR-23a mimics while the luciferase activity of miR-23a NC and pGL3- GAS5-mut was unchanged (Dong Z. et al., 2019). RNA pull-down analysis suggested that approximately 5% of GAS5 bound to miR-23a compared to 100% of GAS5 in total RNA, and these results suggest that miR-23a could pull down GAS5 in liver tissue and HSC. lncRNA GAS5 silencing resulted in increased expression levels of miR-23a while addition of exogenous miR-23a resulted in downregulation of lncRNA GAS5 expression levels, this evidence suggested the ability of lncRNA GAS5 to bind directly to miR-23a. Thus, the ability of lncRNA GAS5 to act as a sponge platform for miR-23a and competitively reduce the expression level of miR-23a to inhibit liver fibrosis can be confirmed. Additionally, it is essential to mention the fact that TCM has been selected as an alternative therapy for liver fibrosis in view of the ineffectiveness and frequent occurrence of adverse side effects of synthetic drugs currently used to treat liver diseases, including liver fibrosis (Lam et al., 2016). Dahuang Zhezhuo Pill (DHZCP) as a typical Chinese medicine can inhibit the proliferation of vascular smooth muscle cells or further development of liver fibrosis *in vivo* by inhibiting the MAPK pathway (Zhang et al., 2009; Cai et al., 2010). Gong et al. identified that the proliferation of HSC was significantly inhibited after overexpression of GAS5 and DHZCP reversed the relative mRNA expression of GAS5, which suggest that DHZCP can mitigate liver fibrosis by enhancing the GAS5 expression (Gong et al., 2018).

### 2.3 LncRNAs Functions as Competitive Endogenous RNAs in Liver Fibrosis

**FIGURE 3:**
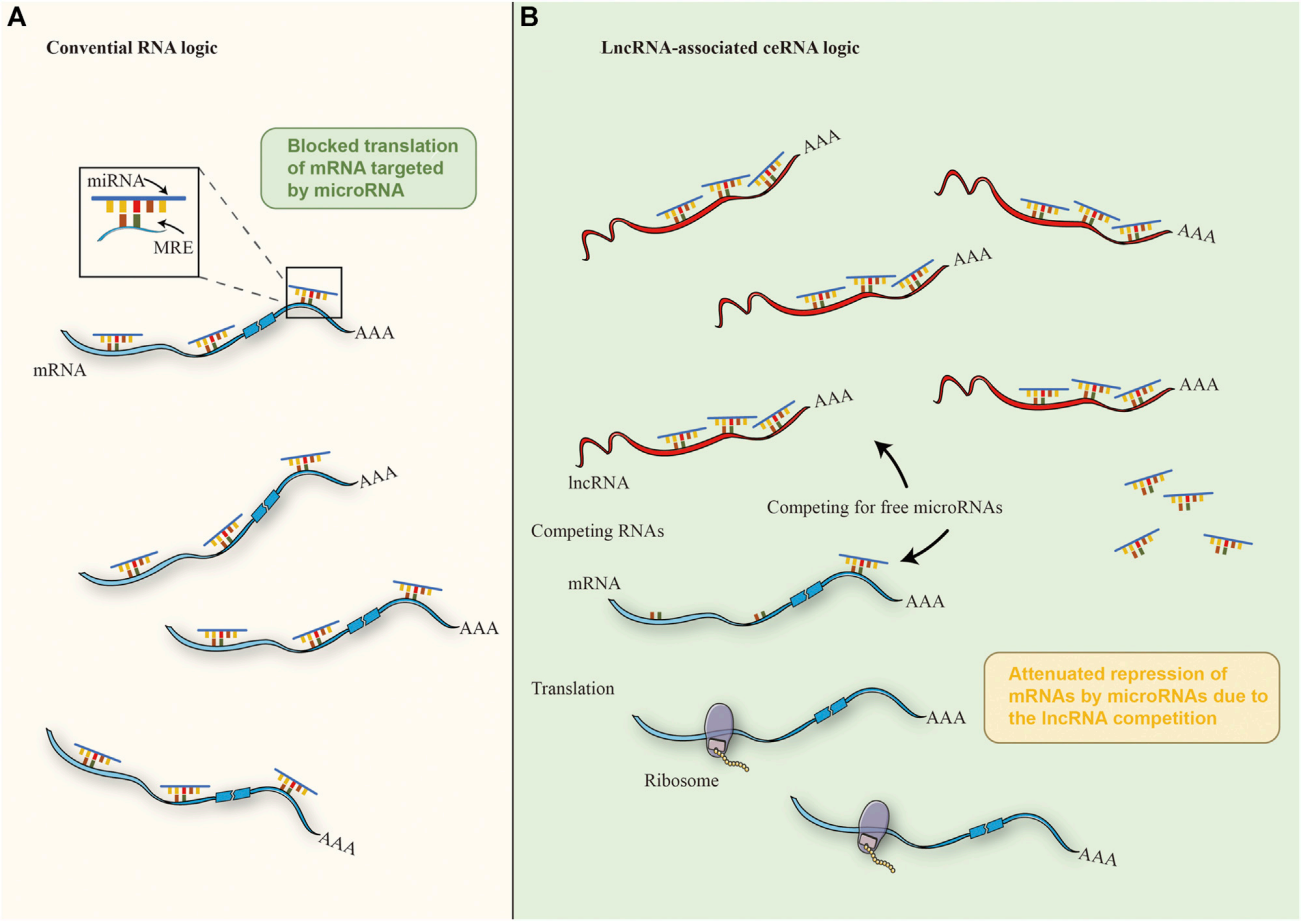
The mechanism of ceRNA. **(A)** In the cytoplasm, miRNAs could regulate 3′- UTR of mRNAs through base pairing with partial complementarity in the conventional crosstalk of RNA transcripts, thus inhibiting mRNAs. **(B)** Under the ceRNA mechanism of cancer cells, miRNAs are isolated from each other by abnormally expressed lncrna and MREs, thus reducing the interaction between miRNA and mRNA, thereby weakening the inhibition of downstream mRNA.

Competitive endogenous RNAs (ceRNAs) act as reciprocal regulators of transcripts at the post-transcriptional level through competing shared miRNAs (Salmena et al., 2011; Qi et al., 2015). ceRNA hypothesis suggests that it provides a pathway to predict the non-coding function of any non-featured RNA transcript by identifying putative miRNA binding sites and linking the function of protein-coding mRNAs to that of e.g., miRNA, lncRNA, and MiRNAs negatively regulate gene expression at the post-transcriptional level by direct base pairing with target sites within the untranslated region of messenger RNAs (Franco-Zorrilla et al., 2007; Thomson and Dinger, 2016; Braga et al., 2020), considering that more than 60% of human protein-coding genes are under the selective pressure of MiRNAs and that any transcript containing miRNA response elements could theoretically function as ceRNAs the ability to function (Salmena et al., 2011; Li et al., 2014; Yang X. et al., 2020), which may typify a wide range of post-transcriptional forms of regulation of gene expression in physiology and pathology (Karreth et al., 2011; An et al., 2017; Wang L. X. et al., 2019). Many lncRNAs may have poor results on their effectiveness as ceRNAs under steady-state conditions due to low abundance and/or nuclear localization. Thousands of lncRNAs have cell type, tissue type, developmental stage, and disease-specific expression patterns and localization suggesting that in some cases individual lncRNAs may be effective natural miRNA sponges (Guttman and Rinn, 2012; Tay et al., 2014) (Figure 3). Preliminary experimental evidence has been given for ceRNA crosstalk results between the tumor suppressor gene PTEN and the pseudogene PTENP1 (Tay et al., 2011), and recent studies have focused on the ability of lncRNAs to act as ceRNAs to regulate miRNA concentrations and biological functions in hepatic fibrosis. using CCl4-induced mice (Zhang et al., 2018; He et al., 2020; Mahpour and Mullen, 2021). Zhu et al. explored that overexpression of H19 significantly exacerbated hepatocyte HSC and EMT activation (Zhu et al., 2019). Dual luciferase reporter analysis mechanistically revealed that miR-148a significantly inhibited the luciferase activity of pmirGLO-H19-WT and deregulated this inhibition by targeted mutation of the binding site. miR-148a inhibitor rescued H19 levels in LX-2 cells but miR-148a mimicked the down-regulated H19 levels in L-02 cells. Overexpression of H19 did not affect miR-148a levels in fibrotic livers but miR-148a could inhibit HSC and EMT activation by targeting ubiquitin-specific protease 4 (USP4) (Zhu et al., 2019). They demonstrated that the maintenance of USP4 levels could be mediated by H19 as ceRNA spongy miR-148a hair, and this evidence suggests that H19 may be a promising target for the treatment of liver fibrosis through the novel H19/miR-148a/USP4 axis that can promote liver fibrosis in HSC and hepatocytes (Zhu et al., 2019). Yu et al. found that liver fibrosis tissue and activation reduced levels of lincRNA-p21 expression in HSC, and overexpression of lincRNA-p21 played a key role in the inhibition of its activation by inducing a significant reduction in HSC expression of α-SMA and Col (Yu et al., 2016). Noticeably, these effects were blocked if in the absence of lincRNA-p21-induced PTEN enhancement, and these circumstances demonstrate the fact that lincRNAp21 inhibits liver fibrosis through PTEN. Further studies showed that miR-181b mimics inhibited the effect of lincRNA-p21 on PTEN expression and HSC activation. Combined with the above data lincRNA-p21 enhances PTEN expression levels by competitively binding miR-181b (Yu et al., 2016). Thus, these results reveal a novel lincRNA-p21-miR-181b-PTEN signaling cascade in liver fibrosis and its potential to suggest lincRNA-p21 as a molecular target for anti-fibrotic therapy. Yu et al. confirmed that lincRNA-p21 inhibits miR-17-5p levels, with this phenomenon missing in lincRNA-p21-miR-17-5p binding site could block miR-17-5p expression in the inhibition assay (Yu et al., 2017a). The function of miR-29b in mediating the downregulation of extracellular matrix genes involved in the TGF-β and NF-κB signaling pathways in HSC has long been reported (Roderburg et al., 2011). Han et al. suggested that TUG1 promotes the expression of these pro-fibrotic genes through the downregulation of miR-29b and thus plays a ceRNA role in accelerating the progression of liver fibrosis (Han et al., 2018). Xie et al. demonstrated that SNHG7 as a ceRNA can also bind to miR-29b in HSC and inhibit the expression level of miR-29b, which may affect the expression of DNMT3A (a downstream target gene of miR-29b) thus regulating the activation, autophagy, and proliferation of HSC (Xie et al., 2021). The downregulation of miR-378a-3p, a target of SNHG7, which is co-localized with SNHG7 in the cytoplasm, could block SNHG7 deletion and thus alleviate the outcome of HSC activation. Analogously, SNHG7-induced HSC activation was almost confirmed to be blocked by irregular fragment polarity protein 2 (DVL2) knockdown of the target site of miR-378a-3p (Yu et al., 2019). These discoveries suggest that lncRNA SNHG7 may interact with different miRNAs to play a critical role in the development of liver fibrosis. Zhou et al. investigated sperm-mediated primary HSC and found that lncRNA Gm5091 overexpressed and knocked downplayed important roles in negatively regulating cell migration, ROS content, IL-1β secretion and HSC activation, respectively (Zhou et al., 2018). lncRNA Gm5091 exhibited direct binding to miR-27b, miR-23b, and miR-24 and inhibited miR-27b, miR-23b, and miR-24 expression. All these suggest the potential of lncRNA Gm5091 to function as ceRNA and thus attenuate liver fibrosis through spongy miR-27b/23b/24 (Zhou et al., 2018). lncRNA ATB containing a common binding site for miR-200a was found to be upregulated in fibrotic liver tissue and simultaneously involved in LX-2 cell activation by Fu et al. in the same field. Knockdown experiments of lncRNA ATB upregulated endogenous miR-200a while downregulating β-catenin expression while suppressing the activation state of LX-2 cells (Fu et al., 2017). Significant increase in lncRNA NEAT1 expression *in vitro* and *in vivo*, as well as the inhibitory effect of its deletion on liver fibrosis were observed (Yu et al., 2017b). lncRNA NEAT1 and miR-122 interacted directly in that lncRNA NEAT1 could regulate KLF6 expression in liver fibrosis by competitively binding to miR-122, thereby accelerating HSC activation and increased cell proliferation and collagen activation (Yu et al., 2017b). The lncRNA NEAT1, which is upregulated in NAFLD progression, binds to miR-506, and GLI3, and regulates GLI3 expression levels as well as fibrosis, inflammatory response and lipid metabolism in NAFLD by secreting miR-506 and miR-506/GLI3 axis, respectively (Jin et al., 2019). lncRNA NEAT1 was found to be elevated in ash by Ye et al. was elevated in ash and acted as a ceRNA sponge for miR-129-5p′s ability to suppress SOCS2 expression. It is also important to note that inhibition of lncRNA NEAT1 inhibits the development of liver fibrosis and ASH by elevating miR-129-5p and inhibiting SOCS2 (Ye et al., 2020). These findings clarify that lncRNA NEAT1 may contribute to the development of liver fibrosis and provide new insights into the pathogenesis and potential therapeutic strategies for liver fibrosis. In conclusion these results suggest that lncRNA-miRNA interactions regulate target genes and play a role in liver fibrosis, and these evidence will provide the basis for a better understanding of this interaction to develop a new liver fibrosis treatment strategy (Table 2).

**TABLE 2:** lncRNA as ceRNA in liver fibrosis.

lncRNAs	miRNA	Mechanism of interaction	Targets	References
lncRNA NEAT1	miR-122/miR-506/miR-129-5p	lncRNA NEAT1 act as sponge of miR-122/miR-506/miR-129-5p	KLF6/GLI3/SOCS2	Yu et al. (2017b); Jin et al. (2019); Ye et al. (2020)
lncRNA SNHG7	miR-29b/miR-378a-3p	lncRNA SNHG7 act as sponge of miR-29b/miR-378a-3p	DNMT3A/DVL2	32893175 Yu et al. (2019); Xie et al. (2021)
lncRNA H19	miR-148a	lncRNA H19 act as sponge of miR-148a	USP4	Zhu et al. (2019)
lncRNA Gm5091	miR-27b/23b/24	lncRNA Gm5091 act as sponge of miR-27b/23b/24	—	Zhou et al. (2018)
lncRNA TUG1	miR-29b	lncRNA TUG1 act as sponge of miR-29b	—	Han et al. (2018)
lincRNA-p21	miR-181b/miR-17-5p	lncRNA lincRNA-p21 act as sponge of miR-181b/miR-17-5p	PTEN/β-catenin	Yu et al. (2016); Yu et al. (2017a)
lncRNA ATB	miR-200a	lncRNA ATB act as sponge of miR-200a	β-catenin	Fu et al. (2017)

## 3 Regulatory Mechanism of lncRNAs in Liver Fibrosis

**FIGURE 4:**
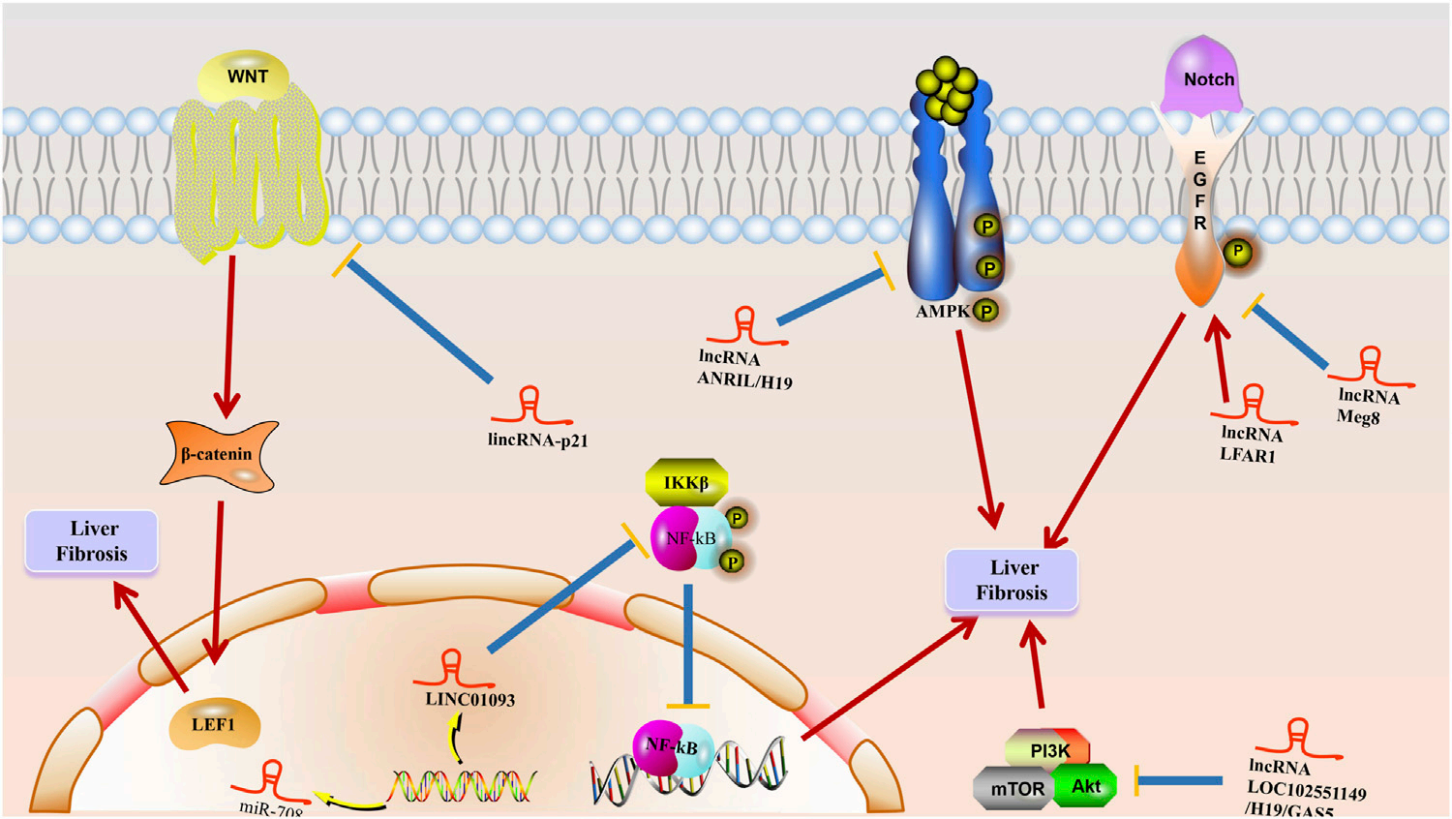
The mechanism of lncRNA to liver fibrosis. Multiple stimuli such as chronic hepatitis B (CHB) damage hepatocytes to initiate wound healing responses, and LncRNAs play a role in promoting activation and apoptosis of hepatic stellate cells and inducing epithelial-mesenchymal transition (EMT) at multiple stages, leading to excessive accumulation of extracellular matrix (ECM) proteins in hepatocytes, resulting in liver fibrosis generation and progression.

Deep understanding of the disease is essential to improve patient survival and to identify effective biomarkers for the development of liver fibrosis. How to detect liver fibrosis early in disease progression and develop effective therapies is critical in reducing the risk of cirrhosis, subsequent decompensation or liver cancer and reducing cancer mortality (Chang et al., 2015; Tacke and Trautwein, 2015; Aydin and Akcali, 2018). We already know that multiple signaling pathways are involved in the pathogenesis of liver fibrosis (Yang et al., 2014; Roehlen et al., 2020; Zhu et al., 2021). We therefore summarize some of the regulatory mechanisms associated with hepatic fibrosis development and progression (Figure 4).

### 3.1 Notch Signaling Pathway

The importance of lncRNAs in mediating various signaling pathways has been recently highlighted in the direction of liver fibrosis onset and progression as well (Peng et al., 2018; Yang et al., 2019; Ganguly and Chakrabarti, 2021). The Notch signaling pathway, which induces developmental interactions and is a major player in liver biology and pathophysiology (Kovall et al., 2017; Nowell and Radtke, 2017; Meurette and Mehlen, 2018), is thought to be involved in cell proliferation, survival, apoptosis and differentiation events at various stages of development thereby controlling events such as organogenesis and morphogenesis (Zhang K. et al., 2019; Chen T. et al., 2020), as well as being significantly associated with HSC activation and HCs EMT in liver fibrosis (Zhang K. et al., 2019). Chen et al. found that lncRNA Meg8, through the Notch pathway inhibited hepatic stellate cell activation and EMT in hepatocytes while its silencing assay exhibited a significant promotion of Notch2, Notch3 and Hes1 expression levels in primary HSC and LX-2 cells (Chen T. et al., 2020). lncRNAs Notch2, Notch3, and Hes1 expression could also be inhibited by knocking down lncRNAs in primary HCs and AML-12 cells Meg8 was significantly increased. Increased mRNA and protein levels of type I collagen and α-SMA were observed in LX-2 cells transfected with lncRNA Meg8 siRNA, while knockdown lncRNA Meg8 experiments showed that overexpression of type I collagen and α-SMA was eliminated by RO4929097, evidence suggesting that this signal may be involved in mediating the function of lncRNA Meg8 (Chen T. et al., 2020). The regulatory role of lncRNAs in liver fibrosis via the Notch signaling pathway was recently reported, and protein and mRNA levels of Notch signaling-related molecules and target genes Notch2, Notch3, and Hes1 were reduced in HSCs with lncRNA LFAR1 downregulation and increased in HSCs with lncRNA LFAR1 overexpression (Zhang K. et al., 2017). CCl4-and BDL-treated mice showed significantly increased expression of Notch2, Notch3, Hes1, and Hey2 compared to lenti NC infection. Lentivirus-mediated knockdown of lncRNA LFAR1 resulted in decreased expression of Notch2, Notch 3, Hes1, and Hey2 while suppressing CCl4-and BDL-induced upregulation of these genes, and this evidence suggests that lncRNA LFAR1 promotes processes such as liver fibrosis and HSC activation through activation of the Notch signaling pathway as well as acting as a Notch signaling pathway provides new insights to elucidate the molecular mechanisms of liver fibrosis (Zhang K. et al., 2017).

### 3.2 Wnt/β-Catenin Signaling Pathway

The Wnt/β-catenin signaling pathway, which is highly conserved among species and controls a variety of biological processes during animal development and life cycle (Zhou and Liu, 2015; Fu et al., 2018; Zuo et al., 2019), is essential in the regulation of EMT and can recur during the onset and progression of various diseases (Sebio et al., 2014; Schunk et al., 2021). Salvianolic acid B (Sal B), one of the water-soluble components extracted from Salvia miltiorrhiza, plays an important role in the treatment and inhibition of activated HSC, and increases the expression of lincRNA-p21 (Yu et al., 2017a). Sal-B increased the expression of P-β-catenin and decreased the cytoplasmic and nuclear expression levels of β-catenin thus significantly reducing the pathway activity while this phenomenon could be restored by lincRNA-p21 knockdown. The deletion of lincRNA-p21 is involved in the inhibition of Sal-B-induced P-β-cateni and the restoration of reduced β-linked proteins in the cytoplasm and nucleus, suggesting that Sal-B may inhibit Wnt/β-linked protein pathway processes through lincRNA-p21 (Yu et al., 2017a). In conclusion, the Wnt/β-linked protein pathway inhibited by lincRNA-p21 is involved in the effect of Sal B on HSC activation and thus inhibits HSC activation and provides new evidence for the role of Wnt/β-linked protein signaling inhibited by lincRNA-p21 in the progression of liver fibrosis disease. proliferation, survival, differentiation, and invasion (Lee JJ. et al., 2015; Alzahrani, 2019; Corti et al., 2019).

### 3.3 PI3K/AKT/mTOR Signaling Pathway

lncRNAs silencing experiments can reduce the phosphorylation levels of ERK, Akt, and mTOR, while PI3K/AKT/mTOR signaling has also been reported to be closely associated with HSC proliferation, activation, and ECM synthesis, which is also significantly inhibited by pharmacological and genetic approaches through inhibition of PI3K signaling (Khemlina et al., 2017; Kong et al., 2020; Jung et al., 2021). Huang et al. revealed that increased expression of H19 could be inhibited by LY294002. These results suggest a role for lncRNA H19 in HSC activation as a downstream site regulated by the PI3K/AKT/mTOR pathway (Huang et al., 2019). Beyond this the pathway of lncRNA H19 promoting HSC activation through autophagy must be highlighted. It has also been reported that lncRNA H19 significantly decreased the expression of *p*-AKT and *p*-mTOR, and this effect was further enhanced by LY294002 and rapamycin (Huang et al., 2019). This suggests that lncRNA H19 can be involved in the PI3K/AKT/mTOR-promoted autophagy-activated HSC pathway 30735452. Dong et al. reported that silencing of lncRNA GAS5 increased the expression levels of p-PI3K, *p*-Akt, and *p*-mTOR thus revealing that activation of PI3K/Akt/mTOR signaling pathway in liver fibrosis can be mediated by the lncRNA GAS5 (Dong Z. et al., 2019). LOC102551149 knockdown assay promoted the expression of p-PI3K, *p*-Akt, and *p*-mTOR in activated HSC, while the overexpression of LOC102551149 in activated HSC 30735452 suppressed the expression levels of p-PI3K, *p*-Akt, and *p*-mTOR (Dong Z. et al., 2019). This evidence imply that lncRNAs can reduce the activation response of HSC by inhibiting the activation of PI3K/AKT/mTOR signaling pathway in liver fibrosis (Karin et al., 2002; De Simone et al., 2015).

### 3.4 NF-κB Signaling Pathway

The NF-κB signaling pathway, an important transcription factor for many inflammatory mediators and cytokines, remains a dormant molecule in the cytoplasm by binding tightly to IκB inhibitor proteins (Inoue et al., 1992; Yang et al., 2012), and phosphorylation of IκB by IκB kinase (IKK) upon stimulation separates IκB from NF-κB leading to translocation and activation of NF-κB, a process reported to be involved in the formation and progression of liver fibrosis (Luedde and Schwabe, 2011; Wang T. et al., 2019; Zhang K. et al., 2020; Zhao et al., 2020). Shi et al. found that LINC01093 31450097 knockdown assay confirmed the promotion of NF-κB p65 nuclear translocation and elevated levels of NF-kB p65 in the cytoplasm (Shi et al., 2019). On the contrary, overexpression of LINC01093 is involved in the inhibition of nuclear translocation of NF-κB p65 leading to an increase in the nuclear level of NF-κB p65 and a decrease in NF-κB p65 at the cytoplasmic level, and this evidence suggests that overexpression of LINC01093 could be involved in inhibiting hepatocyte apoptosis and attenuating the process of liver fibrosis by suppressing the NF-κB signaling pathway (Shi et al., 2019).

### 3.5 AMP-Activated Protein Kinase Signaling Pathway

The AMP-activated protein kinase (AMPK) signaling pathway, which plays an important role in regulating cellular energy homeostasis, could respond to changes in intracellular adenine nucleotide levels and is involved in the process of HSC activation (Shackelford and Shaw, 2009; Mihaylova and Shaw, 2011; Zhao et al., 2017). Yang et al. determined that the proliferation rate of HSC transfected with LncRNA- ANRIL siRNA was significantly higher than that of NC and vector-identified AMPK as a key gene in LncRNA-ANRIL-mediated HSC activation (Zhang T. et al., 2019; Kim MH. et al., 2020). Overexpression of LncRNA-ANRIL suppressed the level of phosphorylated AMPK in activated HSC while LncRNA-ANRIL-siRNA increased the level of phosphorylated AMPK in activated HSC, this evidence suggest that LncRNA-ANRIL deletion can trigger HSC activation through AMPK pathway (Yang JJ. et al., 2020). Wang et al. revealed that lncRNA- H19 regulates lipid droplet metabolism by mechanisms that rely on the AMPKα pathway acting as a sensor for maintaining energy homeostasis (Wang Z. et al., 2020). The upregulated lncRNA-H19 initiates the catabolic pathway by binding to AMPKα to maintain the necessary energy supply. In addition to acting as a scaffold between AMPKα and LKB1, lncRNA-H19 links AMPKα and LKB1 and plays a facilitating role in the phosphorylation of AMPKα by LKB1 (Wang Z. et al., 2020). lncRNA-H19/AMPKα pathway is thought to be involved in HSC activation-induced lipid droplet disappearance in liver fibrosis given that lncRNA-H19 can be observed to induce HSC -formation of the AMPKα/LKB1 complex in LX2 cells and its potential as a novel target for liver fibrosis treatment (Wang Z. et al., 2020).

In conclusion, these findings highlight the possibility that there may be new therapeutic targets and biomarkers for liver fibrosis in the future from lncRNAs, Figure 4 schematically demonstrates the potential mechanism of lncRNA on liver fibrosis.

## 4 Potential Clinical Application of lncRNAs in Human Cancers

As a serious infectious disease caused by hepatitis B virus (HBV) infection, hepatitis B currently infects 350–400 million people worldwide (McMahon, 2009). Patients with chronic hepatitis B (CHB) are characterized by progressive liver fibrosis and inflammation (Guo et al., 2021) as the main pathological manifestations representing the ultimate common pathway for almost all types of CLD (Cai et al., 2020; Roehlen et al., 2020). However, it must be emphasized that liver fibrosis characterized by excessive accumulation of extracellular matrix (ECM) proteins also represents a manifestation of the liver’s trauma healing response to various types of liver injury (e.g., HBV infection) (Lee Y. A. et al., 2015; Tacke and Trautwein, 2015). The application of liver biopsy as the gold standard for assessing the presence and staging of liver fibrosis is often limited by its invasive nature, possible complications, and potential sampling errors. Consequently, there is a need for effective early detection studies of liver fibrosis to control and treat the patient’s liver fibrosis progression (Cadranel et al., 2000; Bravo et al., 2001; Rockey et al., 2009). lncRNAs are frequently deregulated in a variety of human diseases as well as in many important biological processes thereby generating abnormal lncRNAs involved in the development of various diseases 29330108 (Peng et al., 2017; Ma et al., 2018). It must be emphasized that lncRNAs are stable in the circulatory system and readily detectable in serum due to their inability to be degraded by nucleases (Faghihi et al., 2008; Gupta et al., 2010), a property that makes them highly diagnostic in different diseases including liver fibrosis (Zhang K. et al., 2017; Liu et al., 2019). They further demonstrated that reduced serum lincRNA-p21 levels in chronic hepatitis B patients correlated with fibrosis stage (Yu et al., 2017c). Subject operating characteristic curve (ROC) analysis suggested that serum lincRNA-p21 could differentiate chronic hepatitis B patients with liver fibrosis from healthy controls, specifically the area under the ROC curve (AUC) was 0.854 [0.805–0.894], with a sensitivity and specificity of 100 and 70%, respectively, at a critical value of 3.65. A sensitivity of 100% and specificity of 70% accompanied by an AUC of 0.760 (0.682–0.826) in differentiating chronic hepatitis B patients with low fibrosis scores from healthy controls; a sensitivity of 100% and specificity of 73.3% accompanied by an AUC of 0.856 in differentiating chronic hepatitis B patients with moderate fibrosis scores from healthy controls (0.801–0.901); 100% sensitivity and 77.5% specificity accompanied by an AUC of 0.935 (0.882–0.969) were observed in differentiating patients with chronic hepatitis B with high fibrosis score versus healthy controls (Yu et al., 2017c). Furthermore, the levels of lincRNA-p21 could be distinguished in chronic hepatitis B patients with different fibrosis scores, specifically: 70.9% sensitivity and 92.3% specificity (AUC 0.875, 0.800–0.930) for moderate fibrosis score and mild fibrosis score; 81.4% sensitivity and 96.1% specificity (AUC 0.954, 0.859–0.993) for high fibrosis score and low fibrosis score; and Yu et al. showed that serum lincRNA-p21 levels were associated with liver Fibrosis markers including α-SMA and Col1A1 were negatively correlated but markers of viral replication, liver inflammatory activity and liver function showed no correlation (Yu et al., 2017c). lncRNA SNHG7 was also found to be correlated with liver fibrosis progression by Yu et al. (Yu et al., 2019) and ROC curve analysis showed an area under the ROC curve (AUC) of 0.955 (95% confidence interval [CI], 0.868–0.990), where it is noteworthy that at a critical value of 1.0, its sensitivity is 90% and specificity is 100%, suggesting its potential as a potential diagnostic biomarker for liver fibrosis. lncRNA SNHG7 is higher in the cytoplasm of human LX-2 cells as well as primary HSC than in the nucleus (Yu et al., 2019), and this evidence indicates that the expression of lincRNA-p21 and lncRNA SNHG7 plays a key role in the progression of liver fibrosis and its potential as a potential biomarker of liver fibrosis. Han et al. experimentally confirmed that plasma lncRNA GAS5 was significantly elevated in patients with advanced fibrosis compared to patients without progressive fibrosis, but this did not show any statistical difference in tissues, but lncRNA GAS5 tissue expression was positively correlated with the stage of fibrosis prior to the development of cirrhosis as well as significantly downregulating lncRNA in plasma of NAFLD patients with cirrhosis GAS5 expression (Han et al., 2020). However, significant differences in tissue levels of lncRNA GAS5 were not shown in patients with advanced fibrosis and cirrhosis, a phenomenon that emphasizes the accuracy of the association between plasma levels and fibrosis stage. The significance of serum lncRNA GAS5 in the diagnosis of liver fibrosis was proposed by Gou et al. through the detection of abnormalities in lncRNA GAS5 in the serum of patients with chronic hepatitis B, and although the significance of serum lncRNA GAS5 in the age and gender distribution subgroups were not statistically significant (Han et al., 2020). The results of qRT PCR analysis suggested lower serum lncRNA GAS5 levels in CHB patients, and the results of ROC curve analysis showed that serum lncRNA GAS5 could effectively differentiate between CHB liver fibrosis patients and healthy controls (AUC of 0.993, 0.972–0.992). Altogether, circulating elevated lncRNA GAS5 levels correlated with the progression of liver fibrosis prior to the development of cirrhosis can be used to serve as a valid non-invasive marker in patients with NAFLD and CHB with liver fibrosis (Han et al., 2020). Chen et al. contributed significantly to the promotion of lncRNA MEG3 as a serum bi-diagnostic marker for chronic hepatitis B and to improve early diagnosis and treatment outcomes (Chen et al., 2019). qRT PCR data showed a significant decrease in serum lncRNA MEG3 levels in patients with chronic hepatitis B. lncRNA MEG3 expression was negatively correlated with the degree of liver fibrosis (AUC of 0.8844 and the critical value was 5.112) in the low-level fibrosis group versus the control group (AUC and critical value were 0.5237 and 2.988, respectively) (Chen et al., 2019), in the moderate fibrosis group and the control group (AUC and critical value were 0.7085 and 3.812, respectively), and in the high fibrosis group and the control group (AUC and critical value were 0.9395 and 4.689, respectively). Finally, they focused on the possibility of lncRNA MEG3 levels as a differentiating marker in chronic hepatitis B types with different degrees of liver fibrosis (Chen et al., 2019). The AUC and critical values were found to be 0.8281 and 3.963 for the low and intermediate level fibrosis groups, respectively. 0.8857 and 4.818 for the high and low levels fibrosis groups, respectively, and conversely, 0.7861 and 5.312 for the high and intermediate level fibrosis groups, respectively. The results show the important diagnostic value of serum lncRNA MEG3 in patients with chronic hepatitis B combined with liver fibrosis. Yu et al. also concluded that lncRNA MEG3 was negatively correlated with the transcript level of α- SMA and positively correlated with E-calmodulin mRNA expression. Moreover, the increase in fibrosis score was accompanied by a gradual increase in liver MEG3ΔCt value, which indicated that MEG3 expression was negatively correlated with fibrosis score (Yu et al., 2018). In conclusion, all the above results demonstrate that lncRNA MEG3 is a biomarker in the detection and prognosis of liver fibrosis.

Given the current delayed diagnosis and relapse as the biggest barriers to liver fibrosis treatment, ideal biomarkers are of great importance for clinical efforts such as improving early diagnosis rates. These results suggest a potential role of lncRNAs in the diagnosis and prognosis of liver fibrosis. Nevertheless, we must realize that the exact molecular mechanism of the role of lncRNAs in liver fibrosis is still unclear therefore the functional role of lncRNAs in liver fibrosis still needs further exploration and validation including clinical applications.

## 5 Prospects

lncRNAs have been receiving increasing attention along with the rapid development of the field of molecular biology, and breakthroughs in new high-throughput sequencing technologies such as RNA-Seq, microarrays and deep sequencing have provided the basis for expanding our understanding of complex transcriptomic networks and enabling us to identify the dysregulated expression of various lncRNAs in liver fibrosis. Our review details the role of lncRNAs as important regulators in the development of liver fibrosis and the relationship between aberrant lncRNA expression and HSC activation (Yang et al., 2019; De Vincentis et al., 2020). In addition to this, given the increasing number of studies providing data on lncRNAs measured between normal and liver fibrotic tissues, it not only suggests that lncRNAs may be involved in the progression of liver fibrosis but also provides a solid theoretical basis for lncRNAs to become biomarkers for the clinical diagnosis of liver fibrosis (Jiang and Zhang, 2017; Unfried and Fortes, 2020). However, we still need to clarify the regulatory network of lncRNAs in liver fibrosis and the underlying molecular mechanisms are still complex and still inconclusive (Kim YA. et al., 2020; Ganguly and Chakrabarti, 2021). Therefore, the next work should focus on screening effective lncRNAs for the diagnosis and treatment of liver fibrosis and actively promote the development of effective lncRNAs that can be applied in the clinical setting.

On the other hand, a series of lipid bilayer membrane-bound organelles that are released by cells into the environment, which we call “EVs” (Xu et al., 2016), vary in size and could be released from almost all cells under appropriate physiological and pathological conditions (Walker et al., 2019; Mo et al., 2021). One of the hot topics of research is their cargo-carrying function given that their cargo can partially reflect the cellular properties of their origin, exosomes carry significantly different types of RNAs compared to parental cells (Abels and Breakefield, 2016; van Niel et al., 2018). Most importantly, ncRNAs can be shipped in a way that avoids the fate of unprotected ncRNAs that are readily degraded by RNA enzymes in the blood and can furthermore maintain their integrity and activity in circulation (Shen M. et al., 2019; Mori et al., 2019; Hu et al., 2020). Liu et al. found that hepatic lncRNA H19 expression levels correlated with serum exosomal lncRNA H19 levels and severity of liver fibrosis in a mouse model of cholestatic liver injury and in human patients with primary sclerosing cholangitis (PSC) and primary biliary cholangitis (PBC). In contrast, exogenous lncRNA H19 promotes liver fibrosis and enhances the activation and proliferation of HSC (Liu et al., 2019). Our review highlights the status of exogenous lncRNA H19 as a potential diagnostic marker and therapeutic target in the development of cholestatic liver fibrosis, and the potential of targeting these intercellular signaling mechanisms and mediators to increase sensitivity and improve response to conventional therapeutic agents used to treat liver fibrosis and to complement exogenous lncRNAs strategies in liver prevention, diagnosis and treatment. Finally, we must emphasize that gene editing is a technique for targeted modification of DNA nucleotide sequences characterized by the precise severing of targeted DNA fragments and insertion of new gene fragments (Jinek et al., 2012), and that CRISPR/Cas9, which has been successfully applied to the disruption of protein-coding24 sequences in various organisms (Sharma et al., 2021), is a very powerful gene editing tool, and that it also plays a key role in the progression and development of liver fibrosis (Barrangou et al., 2007; Strotskaya et al., 2017). For example, RSPO4-CRISPR applied in a rat model of liver fibrosis showed excellent performance in reducing liver injury and restoring the gut microbiota (Yu et al., 2021). HSC reprogramming via exon-mediated CRISPR/dCas9-VP64 delivery (Luo et al., 2021) has also been reported. Among them it is important to note that lncRNAs have been successfully edited/regulated by the CRISPR/Cas9 system therefore no transgene needs to be introduced (Konermann et al., 2015; Chen B. et al., 2020). Due to its specificity, efficiency, simplicity, and versatility CRISPR/Cas9 has achieved many encouraging successes as a powerful genome engineering tool for the treatment of many diseases including cancer (Goyal et al., 2017; Esposito et al., 2019). For example, the fact that CRISPR/Cas9’s specifically designed GRNA targeting suppresses the upregulated lncRNA UCA1 (uroepithelial carcinoma associated 1) in bladder cancer once again highlights the potential of the CRISPR/Cas9 system for regulating the expression of lncRNAs and for further use as a therapeutic approach in clinical cancer treatment (Yang et al., 2018; Shen M. et al., 2019). Therefore, it is reasonable to assume that CRISPR/Cas9 can regulate the expression of lncRNAs and thus achieve the treatment of liver fibrosis through relevant molecular mechanisms. Importantly, our review provides a summary of the stages by which this budding and maturing technology can be used in the future for drug discovery, cancer therapy, and treatment of other genetic diseases previously considered incurable.

## 6 Conclusion

Research on the involvement of lncRNAs in regulating the development of liver fibrosis are increasing year by year, and the results of *in vivo* and *ex vivo* experiments confirm the significant effect of overexpression and knockdown of lncRNAs in reducing or enhancing the extent of liver fibrosis, suggesting that lncRNAs are promising as new targets for liver fibrosis treatment. The expression of lncRNAs may be a suitable candidate in the issue of potential markers for the diagnosis and prognosis of liver fibrosis. lncRNAs regulate the proliferation, activation and apoptosis of HSC involved in the process of liver fibrosis. The regulation of lncRNAs expression mediated by miRNAs and the inverse regulation of miRNAs expression by lncRNAs are demonstrated. miRNAs are involved in the regulation of lncRNAs expression through sequence-specific binding between them. Multiple molecular mechanisms regulated by lncRNAs including NF-κB signaling pathway are involved in the pathological process of liver fibrosis, while examples of successful implementation of strategies applying regulation of lncRNA expression in preclinical models can already be observed. On the one hand, we can optimistically anticipate the promising clinical applications of therapeutic strategies based on the regulation of lncRNA expression, but on the other hand, we must realize that their safety and reliability still depend on the advancement of knowledge and sophisticated technologies.

## Abbreviations

AMPK, AMP-activated protein kinase; ncRNAs, non-coding RNAs; lncRNAs, Long non-coding RNAs; ceRNAs, competing endogenous RNAs; miRNAs, micro RNAs; ECM, extracellular matrix; NASH, non-alcoholic steatohepatitis; HSCs, hepatic stellate cells; Xist X, chromosome inactivation specific transcript; HULC, hepatocellular carcinoma; NEAT1, Nuclear paraspeckle assembly transcript 1; SNHG7, small nucleolar RNA host gene 7; MALAT1, metastasis-associated lung adenocarcinoma transcript; HOTTIP, HOXA transcript at the distal tip; TUG1, taurine upregulated gene 1; MEG3, maternally expressed gene 3.

## References

[B1] Abels E. R. Breakefield X. O. (2016). Introduction to Extracellular Vesicles: Biogenesis, RNA Cargo Selection, Content, Release, and Uptake. Cell Mol Neurobiol 36 (3), 301–312. 10.1007/s10571-016-0366-z 27053351 PMC5546313

[B2] Alirezaei M. Flynn C. T. Wood M. R. Harkins S. Whitton J. L. (2015). Coxsackievirus Can Exploit LC3 in Both Autophagy-dependent and -independent Manners *In Vivo* . Autophagy 11 (8), 1389–1407. 10.1080/15548627.2015.1063769 26090585 PMC4590631

[B3] Alzahrani A. S. (2019). PI3K/Akt/mTOR Inhibitors in Cancer: At the Bench and Bedside. Semin. Cancer Biol. 59, 125–132. 10.1016/j.semcancer.2019.07.009 31323288

[B4] Amodio N. Stamato M. A. Juli G. Morelli E. Fulciniti M. Manzoni M. (2018). Drugging the lncRNA MALAT1 via LNA gapmeR ASO Inhibits Gene Expression of Proteasome Subunits and Triggers Anti-multiple Myeloma Activity. Leukemia 32 (9), 1948–1957. 10.1038/s41375-018-0067-3 29487387 PMC6127082

[B5] An Y. Furber K. L. Ji S. (2017). Pseudogenes Regulate Parental Gene Expression via ceRNA Network. J. Cel Mol Med 21 (1), 185–192. 10.1111/jcmm.12952 PMC5192809 27561207

[B6] Anwar S. L. Krech T. Hasemeier B. Schipper E. Schweitzer N. Vogel A. (2012). Loss of Imprinting and Allelic Switching at the DLK1-MEG3 Locus in Human Hepatocellular Carcinoma. PLoS One 7 (11), e49462. 10.1371/journal.pone.0049462 23145177 PMC3493531

[B7] Aydin M. M. Akcali K. C. (2018). Liver Fibrosis. Turk J. Gastroenterol. 29 (1), 14–21. 10.5152/tjg.2018.17330 29391303 PMC6322608

[B8] Barrangou R. Fremaux C. Deveau H. Richards M. Boyaval P. Moineau S. (2007). CRISPR Provides Acquired Resistance against Viruses in Prokaryotes. Science 315 (5819), 1709–1712. 10.1126/science.1138140 17379808

[B9] Benetatos L. Dasoula A. Hatzimichael E. Georgiou I. Syrrou M. Bourantas K. L. (2008). Promoter Hypermethylation of the MEG3 (DLK1/MEG3) Imprinted Gene in Multiple Myeloma. Clin. Lymphoma Myeloma 8 (3), 171–175. 10.3816/CLM.2008.n.021 18650181

[B10] Bian E. B. Xiong Z. G. Li J. (2019). New Advances of lncRNAs in Liver Fibrosis, with Specific Focus on lncRNA-miRNA Interactions. J. Cel Physiol 234 (3), 2194–2203. 10.1002/jcp.27069 30229908

[B11] Blaner W. S. O'Byrne S. M. Wongsiriroj N. Kluwe J. D'Ambrosio D. M. Jiang H. (2009). Hepatic Stellate Cell Lipid Droplets: a Specialized Lipid Droplet for Retinoid Storage. Biochim. Biophys. Acta 1791 (6), 467–473. 10.1016/j.bbalip.2008.11.001 19071229 PMC2719539

[B12] Braga E. A. Fridman M. V. Moscovtsev A. A. Filippova E. A. Dmitriev A. A. Kushlinskii N. E. (2020). LncRNAs in Ovarian Cancer Progression, Metastasis, and Main Pathways: ceRNA and Alternative Mechanisms. Int. J. Mol. Sci. 21 (22). 10.3390/ijms21228855 PMC7700431 33238475

[B13] Bravo A. A. Sheth S. G. Chopra S. (2001). Liver Biopsy. N. Engl. J. Med. 344 (7), 495–500. 10.1056/NEJM200102153440706 11172192

[B14] Broerse J. Crassini B. (1984). Investigations of Perception and Imagery Using CAEs: the Role of Experimental Design and Psychophysical Method. Percept Psychophys 35 (2), 155–164. 10.3758/bf03203895 6718212

[B15] Brown J. A. Bulkley D. Wang J. Valenstein M. L. Yario T. A. Steitz T. A. (2014). Structural Insights into the Stabilization of MALAT1 Noncoding RNA by a Bipartite Triple helix. Nat. Struct. Mol. Biol. 21 (7), 633–640. 10.1038/nsmb.2844 24952594 PMC4096706

[B16] Bu F. T. Wang A. Zhu Y. You H. M. Zhang Y. F. Meng X. M. (2020). LncRNA NEAT1: Shedding Light on Mechanisms and Opportunities in Liver Diseases. Liver Int. 40 (11), 2612–2626. 10.1111/liv.14629 32745314

[B17] Cadranel J. F. Rufat P. Degos F. (2000). Practices of Liver Biopsy in France: Results of a Prospective Nationwide Survey. For the Group of Epidemiology of the French Association for the Study of the Liver (AFEF). Hepatology 32 (3), 477–481. 10.1053/jhep.2000.16602 10960438

[B18] Cai B. Dongiovanni P. Corey K. E. Wang X. Shmarakov I. O. Zheng Z. (2020). Macrophage MerTK Promotes Liver Fibrosis in Nonalcoholic Steatohepatitis. Cell Metab 31 (2), 406–e7. e407. 10.1016/j.cmet.2019.11.013 31839486 PMC7004886

[B19] Cai H. Liu X. Zheng J. Xue Y. Ma J. Li Z. (2017). Long Non-coding RNA Taurine Upregulated 1 Enhances Tumor-Induced Angiogenesis through Inhibiting microRNA-299 in Human Glioblastoma. Oncogene 36 (3), 318–331. 10.1038/onc.2016.212 27345398

[B20] Cai H. B. Sun X. G. Liu Z. F. Liu Y. W. Tang J. Liu Q. (2010). Effects of Dahuangzhechong Pills on Cytokines and Mitogen Activated Protein Kinase Activation in Rats with Hepatic Fibrosis. J. Ethnopharmacol 132 (1), 157–164. 10.1016/j.jep.2010.08.019 20723595

[B21] Cavaillé J. Seitz H. Paulsen M. Ferguson-Smith A. C. Bachellerie J. P. (2002). Identification of Tandemly-Repeated C/D snoRNA Genes at the Imprinted Human 14q32 Domain Reminiscent of Those at the Prader-Willi/Angelman Syndrome Region. Hum. Mol. Genet. 11 (13), 1527–1538. 10.1093/hmg/11.13.1527 12045206

[B22] Chang J. Lan T. Li C. Ji X. Zheng L. Gou H. (2015). Activation of Slit2-Robo1 Signaling Promotes Liver Fibrosis. J. Hepatol. 63 (6), 1413–1420. 10.1016/j.jhep.2015.07.033 26264936

[B23] Chaudhry M. A. (2013). Expression Pattern of Small Nucleolar RNA Host Genes and Long Non-coding RNA in X-Rays-Treated Lymphoblastoid Cells. Int. J. Mol. Sci. 14 (5), 9099–9110. 10.3390/ijms14059099 23698766 PMC3676775

[B24] Chen B. Deng S. Ge T. Ye M. Yu J. Lin S. (2020a). Live Cell Imaging and Proteomic Profiling of Endogenous NEAT1 lncRNA by CRISPR/Cas9-mediated Knock-In. Protein Cell 11 (9), 641–660. 10.1007/s13238-020-00706-w 32458346 PMC7452982

[B25] Chen M. Xia Z. Chen C. Hu W. Yuan Y. (2018a). LncRNA MALAT1 Promotes Epithelial-To-Mesenchymal Transition of Esophageal Cancer through Ezh2-Notch1 Signaling Pathway. Anticancer Drugs 29 (8), 767–773. 10.1097/CAD.0000000000000645 29916899

[B26] Chen M. J. Wang X. G. Sun Z. X. Liu X. C. (2019). Diagnostic Value of LncRNA-MEG3 as a Serum Biomarker in Patients with Hepatitis B Complicated with Liver Fibrosis. Eur. Rev. Med. Pharmacol. Sci. 23 (10), 4360–4367. 10.26355/eurrev_201905_17943 31173310

[B27] Chen T. Lin H. Chen X. Li G. Zhao Y. Zheng L. (2020b). LncRNA Meg8 Suppresses Activation of Hepatic Stellate Cells and Epithelial-Mesenchymal Transition of Hepatocytes via the Notch Pathway. Biochem. Biophys. Res. Commun. 521 (4), 921–927. 10.1016/j.bbrc.2019.11.015 31711641

[B28] Chen X. Lun L. Hou H. Tian R. Zhang H. Zhang Y. (2017). The Value of lncRNA HULC as a Prognostic Factor for Survival of Cancer Outcome: A Meta-Analysis. Cell Physiol Biochem 41 (4), 1424–1434. 10.1159/000468005 28315877

[B29] Chen Y. Huang W. Sun W. Zheng B. Wang C. Luo Z. (2018b). LncRNA MALAT1 Promotes Cancer Metastasis in Osteosarcoma via Activation of the PI3K-Akt Signaling Pathway. Cel Physiol Biochem 51 (3), 1313–1326. 10.1159/000495550 30481748

[B30] Chiu H. S. Somvanshi S. Patel E. Chen T. W. Singh V. P. Zorman B. (2018). Pan-Cancer Analysis of lncRNA Regulation Supports Their Targeting of Cancer Genes in Each Tumor Context. Cell Rep 23 (1), 297–e12. e212. 10.1016/j.celrep.2018.03.064 29617668 PMC5906131

[B31] Choudhry H. Albukhari A. Morotti M. Haider S. Moralli D. Smythies J. (2015). Tumor Hypoxia Induces Nuclear Paraspeckle Formation through HIF-2α Dependent Transcriptional Activation of NEAT1 Leading to Cancer Cell Survival. Oncogene 34 (34), 4482–4490. 10.1038/onc.2014.378 25417700 PMC4430310

[B32] Consortium E. P. Birney E. Stamatoyannopoulos J. A. Dutta A. Guigó R. Gingeras T. R. (2007). Identification and Analysis of Functional Elements in 1% of the Human Genome by the ENCODE Pilot Project. Nature 447 (7146), 799–816. 10.1038/nature05874 17571346 PMC2212820

[B33] Corti F. Nichetti F. Raimondi A. Niger M. Prinzi N. Torchio M. (2019). Targeting the PI3K/AKT/mTOR Pathway in Biliary Tract Cancers: A Review of Current Evidences and Future Perspectives. Cancer Treat. Rev. 72, 45–55. 10.1016/j.ctrv.2018.11.001 30476750

[B34] Cui H. Zhang Y. Zhang Q. Chen W. Zhao H. Liang J. (2017). A Comprehensive Genome-wide Analysis of Long Noncoding RNA Expression Profile in Hepatocellular Carcinoma. Cancer Med. 6 (12), 2932–2941. 10.1002/cam4.1180 29047230 PMC5727245

[B35] Dai X. Chen C. Xue J. Xiao T. Mostofa G. Wang D. (2019). Exosomal MALAT1 Derived from Hepatic Cells Is Involved in the Activation of Hepatic Stellate Cells via miRNA-26b in Fibrosis Induced by Arsenite. Toxicol. Lett. 316, 73–84. 10.1016/j.toxlet.2019.09.008 31513886

[B36] De Simone V. Franzè E. Ronchetti G. Colantoni A. Fantini M. C. Di Fusco D. (2015). Th17-type Cytokines, IL-6 and TNF-α Synergistically Activate STAT3 and NF-kB to Promote Colorectal Cancer Cell Growth. Oncogene 34 (27), 3493–3503. 10.1038/onc.2014.286 25174402 PMC4493653

[B37] De Vincentis A. Rahmani Z. Muley M. Vespasiani-Gentilucci U. Ruggiero S. Zamani P. (2020). Long Noncoding RNAs in Nonalcoholic Fatty Liver Disease and Liver Fibrosis: State-Of-The-Art and Perspectives in Diagnosis and Treatment. Drug Discov. Today 25 (7), 1277–1286. 10.1016/j.drudis.2020.05.009 32439605

[B38] Dimitrova N. Zamudio J. R. Jong R. M. Soukup D. Resnick R. Sarma K. (2014). LincRNA-p21 Activates P21 in Cis to Promote Polycomb Target Gene Expression and to Enforce the G1/S Checkpoint. Mol. Cel 54 (5), 777–790. 10.1016/j.molcel.2014.04.025 PMC4103188 24857549

[B39] Ding F. Lai J. Gao Y. Wang G. Shang J. Zhang D. (2019). NEAT1/miR-23a-3p/KLF3: a Novel Regulatory axis in Melanoma Cancer Progression. Cancer Cel Int 19, 217. 10.1186/s12935-019-0927-6 PMC6706883 31462890

[B40] Dodson M. Wani W. Y. Redmann M. Benavides G. A. Johnson M. S. Ouyang X. (2017). Regulation of Autophagy, Mitochondrial Dynamics, and Cellular Bioenergetics by 4-hydroxynonenal in Primary Neurons. Autophagy 13 (11), 1828–1840. 10.1080/15548627.2017.1356948 28837411 PMC5788494

[B41] Dong P. Xiong Y. Yue J. J B Hanley S. Kobayashi N. Todo Y. (2019a). Exploring lncRNA-Mediated Regulatory Networks in Endometrial Cancer Cells and the Tumor Microenvironment: Advances and Challenges. Cancers (Basel) 11 (2), 234. 10.3390/cancers11020234 PMC6406952 30781521

[B42] Dong R. Liu G. B. Liu B. H. Chen G. Li K. Zheng S. (2016). Targeting Long Non-coding RNA-TUG1 Inhibits Tumor Growth and Angiogenesis in Hepatoblastoma. Cell Death Dis 7 (6), e2278. 10.1038/cddis.2016.143 27362796 PMC5108331

[B43] Dong Z. Li S. Wang X. Si L. Ma R. Bao L. (2019b). lncRNA GAS5 Restrains CCl4-Induced Hepatic Fibrosis by Targeting miR-23a through the PTEN/PI3K/Akt Signaling Pathway. Am. J. Physiol. Gastrointest. Liver Physiol. 316 (4), G539–G550. 10.1152/ajpgi.00249.2018 30735452

[B44] Elpek G. Ö. (2014). Cellular and Molecular Mechanisms in the Pathogenesis of Liver Fibrosis: An Update. World J. Gastroenterol. 20 (23), 7260–7276. 10.3748/wjg.v20.i23.7260 24966597 PMC4064072

[B45] Esposito R. Bosch N. Lanzós A. Polidori T. Pulido-Quetglas C. Johnson R. (2019). Hacking the Cancer Genome: Profiling Therapeutically Actionable Long Non-coding RNAs Using CRISPR-Cas9 Screening. Cancer Cell 35 (4), 545–557. 10.1016/j.ccell.2019.01.019 30827888

[B46] Faghihi M. A. Modarresi F. Khalil A. M. Wood D. E. Sahagan B. G. Morgan T. E. (2008). Expression of a Noncoding RNA Is Elevated in Alzheimer's Disease and Drives Rapid Feed-Forward Regulation of Beta-Secretase. Nat. Med. 14 (7), 723–730. 10.1038/nm1784 18587408 PMC2826895

[B47] Fatica A. Bozzoni I. (2014). Long Non-coding RNAs: New Players in Cell Differentiation and Development. Nat. Rev. Genet. 15 (1), 7–21. 10.1038/nrg3606 24296535

[B48] Feng C. Zhao Y. Li Y. Zhang T. Ma Y. Liu Y. (2019). LncRNA MALAT1 Promotes Lung Cancer Proliferation and Gefitinib Resistance by Acting as a miR-200a Sponge. Arch. Bronconeumol 55 (12), 627–633. 10.1016/j.arbres.2019.03.026 31133357

[B49] Feng L. Zhang J. Zhu N. Ding Q. Zhang X. Yu J. (2017). Ubiquitin Ligase SYVN1/HRD1 Facilitates Degradation of the SERPINA1 Z Variant/α-1-Antitrypsin Z Variant via SQSTM1/p62-dependent Selective Autophagy. Autophagy 13 (4), 686–702. 10.1080/15548627.2017.1280207 28121484 PMC5388218

[B50] Franco-Zorrilla J. M. Valli A. Todesco M. Mateos I. Puga M. I. Rubio-Somoza I. (2007). Target Mimicry Provides a New Mechanism for Regulation of microRNA Activity. Nat. Genet. 39 (8), 1033–1037. 10.1038/ng2079 17643101

[B51] Fu N. Zhao S. X. Kong L. B. Du J. H. Ren W. G. Han F. (2017). LncRNA-ATB/microRNA-200a/β-catenin Regulatory axis Involved in the Progression of HCV-Related Hepatic Fibrosis. Gene 618, 1–7. 10.1016/j.gene.2017.03.008 28302418

[B52] Fu X. Zhu X. Qin F. Zhang Y. Lin J. Ding Y. (2018). Linc00210 Drives Wnt/β-Catenin Signaling Activation and Liver Tumor Progression through CTNNBIP1-dependent Manner. Mol. Cancer 17 (1), 73. 10.1186/s12943-018-0783-3 29540185 PMC5853034

[B53] Gabory A. Ripoche M. A. Yoshimizu T. Dandolo L. (2006). The H19 Gene: Regulation and Function of a Non-coding RNA. Cytogenet. Genome Res. 113 (1-4), 188–193. 10.1159/000090831 16575179

[B54] Ganguly N. Chakrabarti S. (2021). Role of Long Non-coding RNAs and R-elated E-pigenetic M-echanisms in L-iver F-ibrosis (Review). Int. J. Mol. Med. 47 (3). 10.3892/ijmm.2021.4856 PMC7846421 33495817

[B55] Garzon R. Liu S. Fabbri M. Liu Z. Heaphy C. E. A. Callegari E. (2009). MicroRNA-29b Induces Global DNA Hypomethylation and Tumor Suppressor Gene Reexpression in Acute Myeloid Leukemia by Targeting Directly DNMT3A and 3B and Indirectly DNMT1. Blood 113 (25), 6411–6418. 10.1182/blood-2008-07-170589 19211935 PMC2710934

[B56] Geerts A. (2001). History, Heterogeneity, Developmental Biology, and Functions of Quiescent Hepatic Stellate Cells. Semin. Liver Dis. 21 (3), 311–335. 10.1055/s-2001-17550 11586463

[B57] Ghafouri-Fard S. Esmaeili M. Taheri M. Samsami M. (2020b). Highly Upregulated in Liver Cancer (HULC): An Update on its Role in Carcinogenesis. J. Cel Physiol 235 (12), 9071–9079. 10.1002/jcp.29765 32372477

[B58] Ghafouri-Fard S. Taheri M. (2019). Maternally Expressed Gene 3 (MEG3): A Tumor Suppressor Long Non Coding RNA. Biomed. Pharmacother. 118, 109129. 10.1016/j.biopha.2019.109129 31326791

[B59] Ghafouri-Fard S. Esmaeili M. Taheri M. (2020a). H19 lncRNA: Roles in Tumorigenesis. Biomed. Pharmacother. 123, 109774. 10.1016/j.biopha.2019.109774 31855739

[B60] Gong Z. Deng C. Xiao H. Peng Y. Hu G. Xiang T. (2018). Effect of Dahuang Zhechong Pills on Long Non-coding RNA Growth Arrest Specific 5 in Rat Models of Hepatic Fibrosis. J. Tradit Chin. Med. 38 (2), 190–196. 10.1016/j.jtcm.2018.04.007 32186058

[B61] Goyal A. Myacheva K. Gross M. Klingenberg M. Duran Arqué B. Diederichs S. (2017). Challenges of CRISPR/Cas9 Applications for Long Non-coding RNA Genes. Nucleic Acids Res. 45 (3), e12. 10.1093/nar/gkw883 28180319 PMC5388423

[B62] Goyal B. Yadav S. R. M. Awasthee N. Gupta S. Kunnumakkara A. B. Gupta S. C. (2021). Diagnostic, Prognostic, and Therapeutic Significance of Long Non-coding RNA MALAT1 in Cancer. Biochim. Biophys. Acta Rev. Cancer 1875 (2), 188502. 10.1016/j.bbcan.2021.188502 33428963

[B63] Guo S. Chen W. Luo Y. Ren F. Zhong T. Rong M. (2015). Clinical Implication of Long Non-coding RNA NEAT1 Expression in Hepatocellular Carcinoma Patients. Int. J. Clin. Exp. Pathol. 8 (5), 5395–5402. 26191242 PMC4503113

[B64] Guo Y. Li C. Zhang R. Zhan Y. Yu J. Tu J. (2021). Epigenetically-regulated Serum GAS5 as a Potential Biomarker for Patients with Chronic Hepatitis B Virus Infection. Cbm 32, 137–146. 10.3233/CBM-203169 PMC12500054 34092613

[B65] Gupta R. A. Shah N. Wang K. C. Kim J. Horlings H. M. Wong D. J. (2010). Long Non-coding RNA HOTAIR Reprograms Chromatin State to Promote Cancer Metastasis. Nature 464 (7291), 1071–1076. 10.1038/nature08975 20393566 PMC3049919

[B66] Guttman M. Donaghey J. Carey B. W. Garber M. Grenier J. K. Munson G. (2011). lincRNAs Act in the Circuitry Controlling Pluripotency and Differentiation. Nature 477 (7364), 295–300. 10.1038/nature10398 21874018 PMC3175327

[B67] Guttman M. Rinn J. L. (2012). Modular Regulatory Principles of Large Non-coding RNAs. Nature 482 (7385), 339–346. 10.1038/nature10887 22337053 PMC4197003

[B68] Han M. H. Lee J. H. Kim G. Lee E. Lee Y. R. Jang S. Y. (2020). Expression of the Long Noncoding RNA GAS5 Correlates with Liver Fibrosis in Patients with Nonalcoholic Fatty Liver Disease. Genes (Basel) 11 (5), 545. 10.3390/genes11050545 PMC7291058 32413995

[B69] Han X. Hong Y. Zhang K. (2018). TUG1 Is Involved in Liver Fibrosis and Activation of HSCs by Regulating miR-29b. Biochem. Biophys. Res. Commun. 503 (3), 1394–1400. 10.1016/j.bbrc.2018.07.054 30017186

[B70] He Y. Wu Y. T. Huang C. Meng X. M. Ma T. T. Wu B. M. (2014). Inhibitory Effects of Long Noncoding RNA MEG3 on Hepatic Stellate Cells Activation and Liver Fibrogenesis. Biochim. Biophys. Acta 1842 (11), 2204–2215. 10.1016/j.bbadis.2014.08.015 25201080

[B71] He Z. Yang D. Fan X. Zhang M. Li Y. Gu X. (2020). The Roles and Mechanisms of lncRNAs in Liver Fibrosis. Int. J. Mol. Sci. 21 (4), 1482. 10.3390/ijms21041482 PMC7073061 32098245

[B72] Heo M. J. Yun J. Kim S. G. (2019). Role of Non-coding RNAs in Liver Disease Progression to Hepatocellular Carcinoma. Arch. Pharm. Res. 42 (1), 48–62. 10.1007/s12272-018-01104-x 30610616

[B73] Houtkooper R. H. Pirinen E. Auwerx J. (2012). Sirtuins as Regulators of Metabolism and Healthspan. Nat. Rev. Mol. Cel Biol 13 (4), 225–238. 10.1038/nrm3293 PMC4872805 22395773

[B74] Hu W. Liu C. Bi Z. Y. Zhou Q. Zhang H. Li L. L. (2020). Comprehensive Landscape of Extracellular Vesicle-Derived RNAs in Cancer Initiation, Progression, Metastasis and Cancer Immunology. Mol. Cancer 19 (1), 102. 10.1186/s12943-020-01199-1 32503543 PMC7273667

[B75] Huang T. J. Ren J. J. Zhang Q. Q. Kong Y. Y. Zhang H. Y. Guo X. H. (2019). IGFBPrP1 Accelerates Autophagy and Activation of Hepatic Stellate Cells via Mutual Regulation between H19 and PI3K/AKT/mTOR Pathway. Biomed. Pharmacother. 116, 109034. 10.1016/j.biopha.2019.109034 31152924

[B76] Huang W. Huang F. Zhang R. Luo H. (2021). LncRNA Neat1 Expedites the Progression of Liver Fibrosis in Mice through Targeting miR-148a-3p and miR-22-3p to Upregulate Cyth3. Cell Cycle 20 (5-6), 490–507. 10.1080/15384101.2021.1875665 33550894 PMC8018424

[B77] Huarte M. Guttman M. Feldser D. Garber M. Koziol M. J. Kenzelmann-Broz D. (2010). A Large Intergenic Noncoding RNA Induced by P53 Mediates Global Gene Repression in the P53 Response. Cell 142 (3), 409–419. 10.1016/j.cell.2010.06.040 20673990 PMC2956184

[B78] Inoue J. Kerr L. D. Kakizuka A. Verma I. M. (1992). I Kappa B Gamma, a 70 Kd Protein Identical to the C-Terminal Half of P110 NF-Kappa B: a New Member of the I Kappa B Family. Cell 68 (6), 1109–1120. 10.1016/0092-8674(92)90082-n 1339305

[B79] Ji P. Diederichs S. Wang W. Böing S. Metzger R. Schneider P. M. (2003). MALAT-1, a Novel Noncoding RNA, and Thymosin Beta4 Predict Metastasis and Survival in Early-Stage Non-small Cell Lung Cancer. Oncogene 22 (39), 8031–8041. 10.1038/sj.onc.1206928 12970751

[B80] Jia M. Jiang L. Wang Y. D. Huang J. Z. Yu M. Xue H. Z. (2016). lincRNA-p21 Inhibits Invasion and Metastasis of Hepatocellular Carcinoma through Notch Signaling-Induced Epithelial-Mesenchymal Transition. Hepatol. Res. 46 (11), 1137–1144. 10.1111/hepr.12659 27391793

[B81] Jiang X. Zhang F. (2017). Long Noncoding RNA: a New Contributor and Potential Therapeutic Target in Fibrosis. Epigenomics 9 (9), 1233–1241. 10.2217/epi-2017-0020 28809130

[B82] Jin S. S. Lin X. F. Zheng J. Z. Wang Q. Guan H. Q. (2019). lncRNA NEAT1 Regulates Fibrosis and Inflammatory Response Induced by Nonalcoholic Fatty Liver by Regulating miR-506/GLI3. Eur. Cytokine Netw. 30 (3), 98–106. 10.1684/ecn.2019.0432 31957704

[B83] Jinek M. Chylinski K. Fonfara I. Hauer M. Doudna J. A. Charpentier E. (2012). A Programmable Dual-RNA-Guided DNA Endonuclease in Adaptive Bacterial Immunity. Science 337 (6096), 816–821. 10.1126/science.1225829 22745249 PMC6286148

[B84] Jung K. Kim M. So J. Lee S. H. Ko S. Shin D. (2021). Farnesoid X Receptor Activation Impairs Liver Progenitor Cell-Mediated Liver Regeneration via the PTEN-Pi3k-AKT-mTOR Axis in Zebrafish. Hepatology 74 (1), 397–410. 10.1002/hep.31679 33314176 PMC8605479

[B85] Jux B. Gosejacob D. Tolksdorf F. Mandel C. Rieck M. Namislo A. (2019). Cytohesin-3 Is Required for Full Insulin Receptor Signaling and Controls Body Weight via Lipid Excretion. Sci. Rep. 9 (1), 3442. 10.1038/s41598-019-40231-3 30837656 PMC6401384

[B86] Kaneko S. Bonasio R. Saldaña-Meyer R. Yoshida T. Son J. Nishino K. (2014). Interactions between JARID2 and Noncoding RNAs Regulate PRC2 Recruitment to Chromatin. Mol. Cel 53 (2), 290–300. 10.1016/j.molcel.2013.11.012 PMC4026005 24374312

[B87] Kapusta A. Kronenberg Z. Lynch V. J. Zhuo X. Ramsay L. Bourque G. (2013). Transposable Elements Are Major Contributors to the Origin, Diversification, and Regulation of Vertebrate Long Noncoding RNAs. Plos Genet. 9 (4), e1003470. 10.1371/journal.pgen.1003470 23637635 PMC3636048

[B88] Karin M. Cao Y. Greten F. R. Li Z. W. (2002). NF-kappaB in Cancer: from Innocent Bystander to Major Culprit. Nat. Rev. Cancer 2 (4), 301–310. 10.1038/nrc780 12001991

[B89] Karreth F. A. Tay Y. Perna D. Ala U. Tan S. M. Rust A. G. (2011). *In Vivo* identification of Tumor- Suppressive PTEN ceRNAs in an Oncogenic BRAF-Induced Mouse Model of Melanoma. Cell 147 (2), 382–395. 10.1016/j.cell.2011.09.032 22000016 PMC3236086

[B90] Katsushima K. Natsume A. Ohka F. Shinjo K. Hatanaka A. Ichimura N. (2016). Targeting the Notch-Regulated Non-coding RNA TUG1 for Glioma Treatment. Nat. Commun. 7, 13616. 10.1038/ncomms13616 27922002 PMC5150648

[B91] Khalil A. M. Guttman M. Huarte M. Garber M. Raj A. Rivea Morales D. (2009). Many Human Large Intergenic Noncoding RNAs Associate with Chromatin-Modifying Complexes and Affect Gene Expression. Proc. Natl. Acad. Sci. U S A. 106 (28), 11667–11672. 10.1073/pnas.0904715106 19571010 PMC2704857

[B92] Khemlina G. Ikeda S. Kurzrock R. (2017). The Biology of Hepatocellular Carcinoma: Implications for Genomic and Immune Therapies. Mol. Cancer 16 (1), 149. 10.1186/s12943-017-0712-x 28854942 PMC5577674

[B93] Khomich O. Ivanov A. V. Bartosch B. (2019). Metabolic Hallmarks of Hepatic Stellate Cells in Liver Fibrosis. Cells 9 (1). 10.3390/cells9010024 PMC7016711 31861818

[B94] Kim J. Piao H. L. Kim B. J. Yao F. Han Z. Wang Y. (2018). Long Noncoding RNA MALAT1 Suppresses Breast Cancer Metastasis. Nat. Genet. 50 (12), 1705–1715. 10.1038/s41588-018-0252-3 30349115 PMC6265076

[B95] Kim M. H. Seong J. B. Huh J. W. Bae Y. C. Lee H. S. Lee D. S. (2020a). Peroxiredoxin 5 Ameliorates Obesity-Induced Non-alcoholic Fatty Liver Disease through the Regulation of Oxidative Stress and AMP-Activated Protein Kinase Signaling. Redox Biol. 28, 101315. 10.1016/j.redox.2019.101315 31505325 PMC6736789

[B96] Kim Y. A. Park K. K. Lee S. J. (2020b). LncRNAs Act as a Link between Chronic Liver Disease and Hepatocellular Carcinoma. Int. J. Mol. Sci. 21 (8), 2883. 10.3390/ijms21082883 PMC7216144 32326098

[B97] Kitagawa M. Kitagawa K. Kotake Y. Niida H. Ohhata T. (2013). Cell Cycle Regulation by Long Non-coding RNAs. Cell Mol Life Sci 70 (24), 4785–4794. 10.1007/s00018-013-1423-0 23880895 PMC3830198

[B98] Klec C. Gutschner T. Panzitt K. Pichler M. (2019). Involvement of Long Non-coding RNA HULC (Highly Up-Regulated in Liver Cancer) in Pathogenesis and Implications for Therapeutic Intervention. Expert Opin. Ther. Targets 23 (3), 177–186. 10.1080/14728222.2019.1570499 30678498

[B99] Konermann S. Brigham M. D. Trevino A. E. Joung J. Abudayyeh O. O. Barcena C. (2015). Genome-scale Transcriptional Activation by an Engineered CRISPR-Cas9 Complex. Nature 517 (7536), 583–588. 10.1038/nature14136 25494202 PMC4420636

[B100] Kong D. Zhang Z. Chen L. Huang W. Zhang F. Wang L. (2020). Curcumin Blunts Epithelial-Mesenchymal Transition of Hepatocytes to Alleviate Hepatic Fibrosis through Regulating Oxidative Stress and Autophagy. Redox Biol. 36, 101600. 10.1016/j.redox.2020.101600 32526690 PMC7287144

[B101] Kong X. Qian X. Duan L. Liu H. Zhu Y. Qi J. (2016). microRNA-372 Suppresses Migration and Invasion by Targeting P65 in Human Prostate Cancer Cells. DNA Cel Biol 35 (12), 828–835. 10.1089/dna.2015.3186 PMC5165676 27673408

[B102] Kopp F. Mendell J. T. (2018). Functional Classification and Experimental Dissection of Long Noncoding RNAs. Cell 172 (3), 393–407. 10.1016/j.cell.2018.01.011 29373828 PMC5978744

[B103] Kovall R. A. Gebelein B. Sprinzak D. Kopan R. (2017). The Canonical Notch Signaling Pathway: Structural and Biochemical Insights into Shape, Sugar, and Force. Dev. Cel 41 (3), 228–241. 10.1016/j.devcel.2017.04.001 PMC5492985 28486129

[B104] Krizhanovsky V. Yon M. Dickins R. A. Hearn S. Simon J. Miething C. (2008). Senescence of Activated Stellate Cells Limits Liver Fibrosis. Cell 134 (4), 657–667. 10.1016/j.cell.2008.06.049 18724938 PMC3073300

[B105] Lam P. Cheung F. Tan H. Y. Wang N. Yuen M. F. Feng Y. (2016). Hepatoprotective Effects of Chinese Medicinal Herbs: A Focus on Anti-inflammatory and Anti-oxidative Activities. Int. J. Mol. Sci. 17 (4), 465. 10.3390/ijms17040465 27043533 PMC4848921

[B106] Lan T. Li C. Yang G. Sun Y. Zhuang L. Ou Y. (2018). Sphingosine Kinase 1 Promotes Liver Fibrosis by Preventing miR-19b-3p-Mediated Inhibition of CCR2. Hepatology 68 (3), 1070–1086. 10.1002/hep.29885 29572892 PMC6174945

[B107] Lee J. J. Loh K. Yap Y. S. (2015a). PI3K/Akt/mTOR Inhibitors in Breast Cancer. Cancer Biol. Med. 12 (4), 342–354. 10.7497/j.issn.2095-3941.2015.0089 26779371 PMC4706528

[B108] Lee Y. A. Wallace M. C. Friedman S. L. (2015b). Pathobiology of Liver Fibrosis: a Translational success story. Gut 64 (5), 830–841. 10.1136/gutjnl-2014-306842 25681399 PMC4477794

[B109] Leti F. Legendre C. Still C. D. Chu X. Petrick A. Gerhard G. S. (2017). Altered Expression of MALAT1 lncRNA in Nonalcoholic Steatohepatitis Fibrosis Regulates CXCL5 in Hepatic Stellate Cells. Transl Res. 190, 25–e21. 10.1016/j.trsl.2017.09.001 28993096 PMC5705449

[B110] Li J. Yang C. Li Y. Chen A. Li L. You Z. (2018a). LncRNA GAS5 Suppresses Ovarian Cancer by Inducing Inflammasome Formation. Biosci. Rep. 38 (2). 10.1042/BSR20171150 PMC5857912 29229673

[B111] Li J. H. Liu S. Zhou H. Qu L. H. Yang J. H. (2014). starBase v2.0: Decoding miRNA-ceRNA, miRNA-ncRNA and Protein-RNA Interaction Networks from Large-Scale CLIP-Seq Data. Nucleic Acids Res. 42, D92–D97. Database issue. 10.1093/nar/gkt1248 24297251 PMC3964941

[B112] Li T. Mo X. Fu L. Xiao B. Guo J. (2016). Molecular Mechanisms of Long Noncoding RNAs on Gastric Cancer. Oncotarget 7 (8), 8601–8612. 10.18632/oncotarget.6926 26788991 PMC4890990

[B113] Li X. Liu R. Huang Z. Gurley E. C. Wang X. Wang J. (2018b). Cholangiocyte-derived Exosomal Long Noncoding RNA H19 Promotes Cholestatic Liver Injury in Mouse and Humans. Hepatology 68 (2), 599–615. 10.1002/hep.29838 29425397 PMC6085159

[B114] Li Y. Zhu J. Tian G. Li N. Li Q. Ye M. (2010). The DNA Methylome of Human Peripheral Blood Mononuclear Cells. Plos Biol. 8 (11), e1000533. 10.1371/journal.pbio.1000533 21085693 PMC2976721

[B115] Li Z. Wang J. Zeng Q. Hu C. Zhang J. Wang H. (2018c). Long Noncoding RNA HOTTIP Promotes Mouse Hepatic Stellate Cell Activation via Downregulating miR-148a. Cel Physiol Biochem 51 (6), 2814–2828. 10.1159/000496012 30562760

[B116] Lin N. Yao Z. Xu M. Chen J. Lu Y. Yuan L. (2019). Long Noncoding RNA MALAT1 Potentiates Growth and Inhibits Senescence by Antagonizing ABI3BP in Gallbladder Cancer Cells. J. Exp. Clin. Cancer Res. 38 (1), 244. 10.1186/s13046-019-1237-5 31174563 PMC6555920

[B117] Lin S. P. Youngson N. Takada S. Seitz H. Reik W. Paulsen M. (2003). Asymmetric Regulation of Imprinting on the Maternal and Paternal Chromosomes at the Dlk1-Gtl2 Imprinted Cluster on Mouse Chromosome 12. Nat. Genet. 35 (1), 97–102. 10.1038/ng1233 12937418

[B118] Liu C. Yang Z. Wu J. Zhang L. Lee S. Shin D. J. (2018). Long Noncoding RNA H19 Interacts with Polypyrimidine Tract-Binding Protein 1 to Reprogram Hepatic Lipid Homeostasis. Hepatology 67 (5), 1768–1783. 10.1002/hep.29654 29140550 PMC5906152

[B119] Liu R. Li X. Zhu W. Wang Y. Zhao D. Wang X. (2019). Cholangiocyte-Derived Exosomal Long Noncoding RNA H19 Promotes Hepatic Stellate Cell Activation and Cholestatic Liver Fibrosis. Hepatology 70 (4), 1317–1335. 10.1002/hep.30662 30985008 PMC6783323

[B120] Luedde T. Schwabe R. F. (2011). NF-kappaB in the Liver-Llinking Injury, Fibrosis, and Hepatocellular Carcinoma. Nat. Rev. Gastroenterol. Hepatol. 8 (2), 108–118. 10.1038/nrgastro.2010.213 21293511 PMC3295539

[B121] Luo N. Li J. Chen Y. Xu Y. Wei Y. Lu J. (2021). Hepatic Stellate Cell Reprogramming via Exosome-Mediated CRISPR/dCas9-VP64 Delivery. Drug Deliv. 28 (1), 10–18. 10.1080/10717544.2020.1850917 33336604 PMC7751418

[B122] Ma Y. Liu L. Yan F. Wei W. Deng J. Sun J. (2016a). Enhanced Expression of Long Non-coding RNA NEAT1 Is Associated with the Progression of Gastric Adenocarcinomas. World J. Surg. Oncol. 14 (1), 41. 10.1186/s12957-016-0799-3 26911892 PMC4765228

[B123] Ma Y. Yang Y. Wang F. Moyer M. P. Wei Q. Zhang P. (2016b). Long Non-coding RNA CCAL Regulates Colorectal Cancer Progression by Activating Wnt/β-Catenin Signalling Pathway via Suppression of Activator Protein 2α. Gut 65 (9), 1494–1504. 10.1136/gutjnl-2014-308392 25994219

[B124] Ma Y. Zhang J. Wen L. Lin A. (2018). Membrane-lipid Associated lncRNA: A New Regulator in Cancer Signaling. Cancer Lett. 419, 27–29. 10.1016/j.canlet.2018.01.008 29330108

[B125] Mahpour A. Mullen A. C. (2021). Our Emerging Understanding of the Roles of Long Non-coding RNAs in normal Liver Function, Disease, and Malignancy. JHEP Rep. 3 (1), 100177. 10.1016/j.jhepr.2020.100177 33294829 PMC7689550

[B126] Mayr B. Montminy M. (2001). Transcriptional Regulation by the Phosphorylation-dependent Factor CREB. Nat. Rev. Mol. Cel Biol 2 (8), 599–609. 10.1038/35085068 11483993

[B127] McMahon B. J. (2009). The Natural History of Chronic Hepatitis B Virus Infection. Hepatology 49 (5 Suppl. l), S45–S55. 10.1002/hep.22898 19399792

[B128] Mercer T. R. Dinger M. E. Sunkin S. M. Mehler M. F. Mattick J. S. (2008). Specific Expression of Long Noncoding RNAs in the Mouse Brain. Proc. Natl. Acad. Sci. U S A. 105 (2), 716–721. 10.1073/pnas.0706729105 18184812 PMC2206602

[B129] Meurette O. Mehlen P. (2018). Notch Signaling in the Tumor Microenvironment. Cancer Cell 34 (4), 536–548. 10.1016/j.ccell.2018.07.009 30146333

[B130] Mihaylova M. M. Shaw R. J. (2011). The AMPK Signalling Pathway Coordinates Cell Growth, Autophagy and Metabolism. Nat. Cel Biol 13 (9), 1016–1023. 10.1038/ncb2329 PMC3249400 21892142

[B131] Mo Z. Cheong J. Y. A. Xiang L. Le M. T. N. Grimson A. Zhang D. X. (2021). Extracellular Vesicle-Associated Organotropic Metastasis. Cell Prolif 54 (1), e12948. 10.1111/cpr.12948 33145869 PMC7791170

[B132] Mondal T. Subhash S. Vaid R. Enroth S. Uday S. Reinius B. (2015). MEG3 Long Noncoding RNA Regulates the TGF-β Pathway Genes through Formation of RNA-DNA Triplex Structures. Nat. Commun. 6, 7743. 10.1038/ncomms8743 26205790 PMC4525211

[B133] Monti-Rocha R. Cramer A. Gaio Leite P. Antunes M. M. Pereira R. V. S. Barroso A. (2018). SOCS2 Is Critical for the Balancing of Immune Response and Oxidate Stress Protecting against Acetaminophen-Induced Acute Liver Injury. Front. Immunol. 9, 3134. 10.3389/fimmu.2018.03134 30723477 PMC6349694

[B134] Mori M. A. Ludwig R. G. Garcia-Martin R. Brandão B. B. Kahn C. R. (2019). Extracellular miRNAs: From Biomarkers to Mediators of Physiology and Disease. Cel Metab 30 (4), 656–673. 10.1016/j.cmet.2019.07.011 PMC6774861 31447320

[B135] Murphy S. K. Wylie A. A. Coveler K. J. Cotter P. D. Papenhausen P. R. Sutton V. R. (2003). Epigenetic Detection of Human Chromosome 14 Uniparental Disomy. Hum. Mutat. 22 (1), 92–97. 10.1002/humu.10237 12815599

[B136] Ni W. Yao S. Zhou Y. Liu Y. Huang P. Zhou A. (2019). Long Noncoding RNA GAS5 Inhibits Progression of Colorectal Cancer by Interacting with and Triggering YAP Phosphorylation and Degradation and Is Negatively Regulated by the m6A Reader YTHDF3. Mol. Cancer 18 (1), 143. 10.1186/s12943-019-1079-y 31619268 PMC6794841

[B137] Nielsen C. P. Jernigan K. K. Diggins N. L. Webb D. J. MacGurn J. A. (2019). USP9X Deubiquitylates DVL2 to Regulate WNT Pathway Specification. Cel Rep 28 (4), 1074–e5. e1075. 10.1016/j.celrep.2019.06.083 PMC6884140 31340145

[B138] Niland C. N. Merry C. R. Khalil A. M. (2012). Emerging Roles for Long Non-coding RNAs in Cancer and Neurological Disorders. Front. Genet. 3, 25. 10.3389/fgene.2012.00025 22375145 PMC3286759

[B139] Nowell C. S. Radtke F. (2017). Notch as a Tumour Suppressor. Nat. Rev. Cancer 17 (3), 145–159. 10.1038/nrc.2016.145 28154375

[B140] Nudelman R. Ardon O. Hadar Y. Chen Y. Libman J. Shanzer A. (1998). Modular Fluorescent-Labeled Siderophore Analogues. J. Med. Chem. 41 (10), 1671–1678. 10.1021/jm970581b 9572892

[B141] Özgür E. Mert U. Isin M. Okutan M. Dalay N. Gezer U. (2013). Differential Expression of Long Non-coding RNAs during Genotoxic Stress-Induced Apoptosis in HeLa and MCF-7 Cells. Clin. Exp. Med. 13 (2), 119–126. 10.1007/s10238-012-0181-x 22487937

[B142] Page A. Paoli P. Moran Salvador E. White S. French J. Mann J. (2016). Hepatic Stellate Cell Transdifferentiation Involves Genome-wide Remodeling of the DNA Methylation Landscape. J. Hepatol. 64 (3), 661–673. 10.1016/j.jhep.2015.11.024 26632634 PMC4904781

[B143] Panzitt K. Tschernatsch M. M. Guelly C. Moustafa T. Stradner M. Strohmaier H. M. (2007). Characterization of HULC, a Novel Gene with Striking Up-Regulation in Hepatocellular Carcinoma, as Noncoding RNA. Gastroenterology 132 (1), 330–342. 10.1053/j.gastro.2006.08.026 17241883

[B144] Parasramka M. A. Maji S. Matsuda A. Yan I. K. Patel T. (2016). Long Non-coding RNAs as Novel Targets for Therapy in Hepatocellular Carcinoma. Pharmacol. Ther. 161, 67–78. 10.1016/j.pharmthera.2016.03.004 27013343 PMC4851900

[B145] Parola M. Pinzani M. (2019). Liver Fibrosis: Pathophysiology, Pathogenetic Targets and Clinical Issues. Mol. Aspects Med. 65, 37–55. 10.1016/j.mam.2018.09.002 30213667

[B146] Peng H. Wan L. Y. Liang J. J. Zhang Y. Q. Ai W. B. Wu J. F. (2018). The Roles of lncRNA in Hepatic Fibrosis. Cell Biosci 8, 63. 10.1186/s13578-018-0259-6 30534359 PMC6282372

[B147] Peng W. X. Koirala P. Mo Y. Y. (2017). LncRNA-mediated Regulation of Cell Signaling in Cancer. Oncogene 36 (41), 5661–5667. 10.1038/onc.2017.184 28604750 PMC6450570

[B148] Qi X. Zhang D. H. Wu N. Xiao J. H. Wang X. Ma W. (2015). ceRNA in Cancer: Possible Functions and Clinical Implications. J. Med. Genet. 52 (10), 710–718. 10.1136/jmedgenet-2015-103334 26358722

[B149] Quagliata L. Matter M. S. Piscuoglio S. Arabi L. Ruiz C. Procino A. (2014). Long Noncoding RNA HOTTIP/HOXA13 Expression Is Associated with Disease Progression and Predicts Outcome in Hepatocellular Carcinoma Patients. Hepatology 59 (3), 911–923. 10.1002/hep.26740 24114970 PMC3943759

[B150] Raveh E. Matouk I. J. Gilon M. Hochberg A. (2015). The H19 Long Non-coding RNA in Cancer Initiation, Progression and Metastasis - a Proposed Unifying Theory. Mol. Cancer 14, 184. 10.1186/s12943-015-0458-2 26536864 PMC4632688

[B151] Ray K. (2014). Liver: Hepatic Stellate Cells Hold the Key to Liver Fibrosis. Nat. Rev. Gastroenterol. Hepatol. 11 (2), 74. 10.1038/nrgastro.2013.244 24322897

[B152] Riaz F. Li D. (2019). Non-coding RNA Associated Competitive Endogenous RNA Regulatory Network: Novel Therapeutic Approach in Liver Fibrosis. Curr. Gene Ther. 19 (5), 305–317. 10.2174/1566523219666191107113046 31696817

[B153] Rockey D. C. Caldwell S. H. Goodman Z. D. Nelson R. C. Smith A. D. American Association for the Study of Liver Diseases (2009). Liver Biopsy. Hepatology 49 (3), 1017–1044. 10.1002/hep.22742 19243014

[B154] Roderburg C. Urban G. W. Bettermann K. Vucur M. Zimmermann H. Schmidt S. (2011). Micro-RNA Profiling Reveals a Role for miR-29 in Human and Murine Liver Fibrosis. Hepatology 53 (1), 209–218. 10.1002/hep.23922 20890893

[B155] Roehlen N. Crouchet E. Baumert T. F. (2020). Liver Fibrosis: Mechanistic Concepts and Therapeutic Perspectives. Cells 9 (4), 875. 10.3390/cells9040875 PMC7226751 32260126

[B156] Salmena L. Poliseno L. Tay Y. Kats L. Pandolfi P. P. (2011). A ceRNA Hypothesis: the Rosetta Stone of a Hidden RNA Language? Cell 146 (3), 353–358. 10.1016/j.cell.2011.07.014 21802130 PMC3235919

[B157] Schunk S. J. Floege J. Fliser D. Speer T. (2021). WNT-β-catenin Signalling - a Versatile Player in Kidney Injury and Repair. Nat. Rev. Nephrol. 17 (3), 172–184. 10.1038/s41581-020-00343-w 32989282

[B158] Sebio A. Kahn M. Lenz H. J. (2014). The Potential of Targeting Wnt/β-Catenin in colon Cancer. Expert Opin. Ther. Targets 18 (6), 611–615. 10.1517/14728222.2014.906580 24702624

[B159] Shackelford D. B. Shaw R. J. (2009). The LKB1-AMPK Pathway: Metabolism and Growth Control in Tumour Suppression. Nat. Rev. Cancer 9 (8), 563–575. 10.1038/nrc2676 19629071 PMC2756045

[B160] Sharma G. Sharma A. R. Bhattacharya M. Lee S. S. Chakraborty C. (2021). CRISPR-Cas9: A Preclinical and Clinical Perspective for the Treatment of Human Diseases. Mol. Ther. 29 (2), 571–586. 10.1016/j.ymthe.2020.09.028 33238136 PMC7854284

[B161] Shen M. Dong C. Ruan X. Yan W. Cao M. Pizzo D. (2019a). Chemotherapy-Induced Extracellular Vesicle MiRNAs Promote Breast Cancer Stemness by Targeting ONECUT2. Cancer Res. 79 (14), 3608–3621. 10.1158/0008-5472.CAN-18-4055 31118200 PMC8972808

[B162] Shen X. Guo H. Xu J. Wang J. (2019b). Inhibition of lncRNA HULC Improves Hepatic Fibrosis and Hepatocyte Apoptosis by Inhibiting the MAPK Signaling Pathway in Rats with Nonalcoholic Fatty Liver Disease. J. Cel Physiol 234 (10), 18169–18179. 10.1002/jcp.28450 30908654

[B163] Shi X. Jiang X. Yuan B. Liu T. Tang Y. Che Y. (2019). LINC01093 Upregulation Protects against Alcoholic Hepatitis through Inhibition of NF-Κb Signaling Pathway. Mol. Ther. Nucleic Acids 17, 791–803. 10.1016/j.omtn.2019.06.018 31450097 PMC6716105

[B164] Song Y. Liu C. Liu X. Trottier J. Beaudoin M. Zhang L. (2017). H19 Promotes Cholestatic Liver Fibrosis by Preventing ZEB1-Mediated Inhibition of Epithelial Cell Adhesion Molecule. Hepatology 66 (4), 1183–1196. 10.1002/hep.29209 28407375 PMC5605402

[B165] Strotskaya A. Savitskaya E. Metlitskaya A. Morozova N. Datsenko K. A. Semenova E. (2017). The Action of *Escherichia coli* CRISPR-Cas System on Lytic Bacteriophages with Different Lifestyles and Development Strategies. Nucleic Acids Res. 45 (4), 1946–1957. 10.1093/nar/gkx042 28130424 PMC5389539

[B166] Sun D. Yu Z. Fang X. Liu M. Pu Y. Shao Q. (2017). LncRNA GAS5 Inhibits Microglial M2 Polarization and Exacerbates Demyelination. EMBO Rep. 18 (10), 1801–1816. 10.15252/embr.201643668 28808113 PMC5623836

[B167] Tacke F. Trautwein C. (2015). Mechanisms of Liver Fibrosis Resolution. J. Hepatol. 63 (4), 1038–1039. 10.1016/j.jhep.2015.03.039 26232376

[B168] Tang S. S. Zheng B. Y. Xiong X. D. (2015). LincRNA-p21: Implications in Human Diseases. Int. J. Mol. Sci. 16 (8), 18732–18740. 10.3390/ijms160818732 26270659 PMC4581268

[B169] Tay Y. Kats L. Salmena L. Weiss D. Tan S. M. Ala U. (2011). Coding-independent Regulation of the Tumor Suppressor PTEN by Competing Endogenous mRNAs. Cell 147 (2), 344–357. 10.1016/j.cell.2011.09.029 22000013 PMC3235920

[B170] Tay Y. Rinn J. Pandolfi P. P. (2014). The Multilayered Complexity of ceRNA Crosstalk and Competition. Nature 505 (7483), 344–352. 10.1038/nature12986 24429633 PMC4113481

[B171] Thomson D. W. Dinger M. E. (2016). Endogenous microRNA Sponges: Evidence and Controversy. Nat. Rev. Genet. 17 (5), 272–283. 10.1038/nrg.2016.20 27040487

[B172] Tian X. Xu G. (2015). Clinical Value of lncRNA MALAT1 as a Prognostic Marker in Human Cancer: Systematic Review and Meta-Analysis. BMJ Open 5 (9), e008653. 10.1136/bmjopen-2015-008653 PMC4593150 26423854

[B173] Tierling S. Dalbert S. Schoppenhorst S. Tsai C. E. Oliger S. Ferguson-Smith A. C. (2006). High-resolution Map and Imprinting Analysis of the Gtl2-Dnchc1 Domain on Mouse Chromosome 12. Genomics 87 (2), 225–235. 10.1016/j.ygeno.2005.09.018 16309881

[B174] Tsang F. H. Au S. L. Wei L. Fan D. N. Lee J. M. Wong C. C. (2015). Long Non-coding RNA HOTTIP Is Frequently Up-Regulated in Hepatocellular Carcinoma and Is Targeted by Tumour Suppressive miR-125b. Liver Int. 35 (5), 1597–1606. 10.1111/liv.12746 25424744

[B175] Tu X. Zhang Y. Zheng X. Deng J. Li H. Kang Z. (2017). TGF-β-induced Hepatocyte lincRNA-P21 Contributes to Liver Fibrosis in Mice. Sci. Rep. 7 (1), 2957. 10.1038/s41598-017-03175-0 28592847 PMC5462818

[B176] Unfried J. P. Fortes P. (2020). LncRNAs in HCV Infection and HCV-Related Liver Disease. Int. J. Mol. Sci. 21 (6). 10.3390/ijms21062255 PMC7139329 32214045

[B177] van Kruijsbergen I. Hontelez S. Veenstra G. J. (2015). Recruiting Polycomb to Chromatin. Int. J. Biochem. Cel Biol 67, 177–187. 10.1016/j.biocel.2015.05.006 PMC4564301 25982201

[B178] van Niel G. D'Angelo G. Raposo G. (2018). Shedding Light on the Cell Biology of Extracellular Vesicles. Nat. Rev. Mol. Cel Biol 19 (4), 213–228. 10.1038/nrm.2017.125 29339798

[B179] Voce D. J. Bernal G. M. Wu L. Crawley C. D. Zhang W. Mansour N. M. (2019). Temozolomide Treatment Induces lncRNA MALAT1 in an NF-Κb and P53 Codependent Manner in Glioblastoma. Cancer Res. 79 (10), 2536–2548. 10.1158/0008-5472.CAN-18-2170 30940658 PMC6522287

[B180] Walker S. Busatto S. Pham A. Tian M. Suh A. Carson K. (2019). Extracellular Vesicle-Based Drug Delivery Systems for Cancer Treatment. Theranostics 9 (26), 8001–8017. 10.7150/thno.37097 31754377 PMC6857056

[B181] Wang F. Yuan J. H. Wang S. B. Yang F. Yuan S. X. Ye C. (2014). Oncofetal Long Noncoding RNA PVT1 Promotes Proliferation and Stem Cell-like Property of Hepatocellular Carcinoma Cells by Stabilizing NOP2. Hepatology 60 (4), 1278–1290. 10.1002/hep.27239 25043274

[B182] Wang J. Liu X. Wu H. Ni P. Gu Z. Qiao Y. (2010). CREB Up-Regulates Long Non-coding RNA, HULC Expression through Interaction with microRNA-372 in Liver Cancer. Nucleic Acids Res. 38 (16), 5366–5383. 10.1093/nar/gkq285 20423907 PMC2938198

[B183] Wang J. Sun J. Yang F. (2020a). The Role of Long Non-coding RNA H19 in Breast Cancer. Oncol. Lett. 19 (1), 7–16. 10.3892/ol.2019.11093 31897110 PMC6924119

[B184] Wang K. C. Yang Y. W. Liu B. Sanyal A. Corces-Zimmerman R. Chen Y. (2011). A Long Noncoding RNA Maintains Active Chromatin to Coordinate Homeotic Gene Expression. Nature 472 (7341), 120–124. 10.1038/nature09819 21423168 PMC3670758

[B185] Wang L. X. Wan C. Dong Z. B. Wang B. H. Liu H. Y. Li Y. (2019a). Integrative Analysis of Long Noncoding RNA (lncRNA), microRNA (miRNA) and mRNA Expression and Construction of a Competing Endogenous RNA (ceRNA) Network in Metastatic Melanoma. Med. Sci. Monit. 25, 2896–2907. 10.12659/MSM.913881 31004080 PMC6487673

[B186] Wang P. Wu T. Zhou H. Jin Q. He G. Yu H. (2016). Long Noncoding RNA NEAT1 Promotes Laryngeal Squamous Cell Cancer through Regulating miR-107/CDK6 Pathway. J. Exp. Clin. Cancer Res. 35, 22. 10.1186/s13046-016-0297-z 26822763 PMC4731996

[B187] Wang T. Fu X. Jin T. Zhang L. Liu B. Wu Y. (2019b). Aspirin Targets P4HA2 through Inhibiting NF-Κb and LMCD1-AS1/let-7g to Inhibit Tumour Growth and Collagen Deposition in Hepatocellular Carcinoma. EBioMedicine 45, 168–180. 10.1016/j.ebiom.2019.06.048 31278071 PMC6642319

[B188] Wang X. Ruan Y. Wang X. Zhao W. Jiang Q. Jiang C. (2017). Long Intragenic Non-coding RNA lincRNA-P21 Suppresses Development of Human Prostate Cancer. Cel Prolif 50 (2). 10.1111/cpr.12318 PMC6529152 27976428

[B189] Wang Z. Yang X. Kai J. Wang F. Wang Z. Shao J. (2020b). HIF-1α-upregulated lncRNA-H19 Regulates Lipid Droplet Metabolism through the AMPKα Pathway in Hepatic Stellate Cells. Life Sci. 255, 117818. 10.1016/j.lfs.2020.117818 32445757

[B190] Wang Z. M. Xia S. W. Zhang T. Wang Z. Y. Yang X. Kai J. (2020c). LncRNA-H19 Induces Hepatic Stellate Cell Activation via Upregulating Alcohol Dehydrogenase III-Mediated Retinoic Acid Signals. Int. Immunopharmacol 84, 106470. 10.1016/j.intimp.2020.106470 32304991

[B191] Wei L. Q. Li L. Lu C. Liu J. Chen Y. Wu H. (2019a). Involvement of H19/miR-326 axis in Hepatocellular Carcinoma Development through Modulating TWIST1. J. Cel Physiol 234 (4), 5153–5162. 10.1002/jcp.27319 30362512

[B192] Wei S. Wang K. Huang X. Zhao Z. Zhao Z. (2019b). LncRNA MALAT1 Contributes to Non-small Cell Lung Cancer Progression via Modulating miR-200a-3p/programmed Death-Ligand 1 axis. Int. J. Immunopathol Pharmacol. 33, 2058738419859699. 10.1177/2058738419859699 31240979 PMC6595645

[B193] Wilusz C. J. Wilusz J. (2012). HuR and Translation-Tthe Missing Linc(RNA). Mol. Cel 47 (4), 495–496. 10.1016/j.molcel.2012.08.005 22920290

[B194] Wilusz J. E. Freier S. M. Spector D. L. (2008). 3' End Processing of a Long Nuclear-Retained Noncoding RNA Yields a tRNA-like Cytoplasmic RNA. Cell 135 (5), 919–932. 10.1016/j.cell.2008.10.012 19041754 PMC2722846

[B195] Wilusz J. E. (2016). Long Noncoding RNAs: Re-writing Dogmas of RNA Processing and Stability. Biochim. Biophys. Acta 1859 (1), 128–138. 10.1016/j.bbagrm.2015.06.003 26073320 PMC4676738

[B196] Wirawan E. Lippens S. Vanden Berghe T. Romagnoli A. Fimia G. M. Piacentini M. (2012). Beclin1: a Role in Membrane Dynamics and beyond. Autophagy 8 (1), 6–17. 10.4161/auto.8.1.16645 22170155

[B197] Wu F. Sui Y. Wang Y. Xu T. Fan L. Zhu H. (2020a). Long Noncoding RNA SNHG7, a Molecular Sponge for microRNA-485, Promotes the Aggressive Behavior of Cervical Cancer by Regulating PAK4. Onco Targets Ther. 13, 685–699. 10.2147/OTT.S232542 32158221 PMC6986251

[B198] Wu G. Cai J. Han Y. Chen J. Huang Z. P. Chen C. (2014). LincRNA-p21 Regulates Neointima Formation, Vascular Smooth Muscle Cell Proliferation, Apoptosis, and Atherosclerosis by Enhancing P53 Activity. Circulation 130 (17), 1452–1465. 10.1161/CIRCULATIONAHA.114.011675 25156994 PMC4244705

[B199] Wu X. Yuan Y. Ma R. Xu B. Zhang R. (2020b). lncRNA SNHG7 Affects Malignant Tumor Behaviors through Downregulation of EZH2 in Uveal Melanoma Cell Lines. Oncol. Lett. 19 (2), 1505–1515. 10.3892/ol.2019.11240 32002036 PMC6960395

[B200] Wu Y. Liu X. Zhou Q. Huang C. Meng X. Xu F. (2015). Silent Information Regulator 1 (SIRT1) Ameliorates Liver Fibrosis via Promoting Activated Stellate Cell Apoptosis and Reversion. Toxicol. Appl. Pharmacol. 289 (2), 163–176. 10.1016/j.taap.2015.09.028 26435214

[B201] Wylie A. A. Murphy S. K. Orton T. C. Jirtle R. L. (2000). Novel Imprinted DLK1/GTL2 Domain on Human Chromosome 14 Contains Motifs that Mimic Those Implicated in IGF2/H19 Regulation. Genome Res. 10 (11), 1711–1718. 10.1101/gr.161600 11076856 PMC310985

[B202] Xia Q. Li J. Yang Z. Zhang D. Tian J. Gu B. (2020). Long Non-coding RNA Small Nucleolar RNA Host Gene 7 Expression Level in Prostate Cancer Tissues Predicts the Prognosis of Patients with Prostate Cancer. Medicine (Baltimore) 99 (7), e18993. 10.1097/MD.0000000000018993 32049793 PMC7035107

[B203] Xiao Y. Liu R. Li X. Gurley E. C. Hylemon P. B. Lu Y. (2019). Long Noncoding RNA H19 Contributes to Cholangiocyte Proliferation and Cholestatic Liver Fibrosis in Biliary Atresia. Hepatology 70 (5), 1658–1673. 10.1002/hep.30698 31063660 PMC6819224

[B204] Xie J. J. Li W. H. Li X. Ye W. Shao C. F. (2019). LncRNA MALAT1 Promotes Colorectal Cancer Development by Sponging miR-363-3p to Regulate EZH2 Expression. J. Biol. Regul. Homeost Agents 33 (2), 331–343. 30972996

[B205] Xie Z. Wu Y. Liu S. Lai Y. Tang S. (2021). LncRNA-SNHG7/miR-29b/DNMT3A axis Affects Activation, Autophagy and Proliferation of Hepatic Stellate Cells in Liver Fibrosis. Clin. Res. Hepatol. Gastroenterol. 45 (2), 101469. 10.1016/j.clinre.2020.05.017 32893175

[B206] Xin X. Wu M. Meng Q. Wang C. Lu Y. Yang Y. (2018). Long Noncoding RNA HULC Accelerates Liver Cancer by Inhibiting PTEN via Autophagy Cooperation to miR15a. Mol. Cancer 17 (1), 94. 10.1186/s12943-018-0843-8 29895332 PMC5998602

[B207] Xiong D. Sheng Y. Ding S. Chen J. Tan X. Zeng T. (2016). LINC00052 Regulates the Expression of NTRK3 by miR-128 and miR-485-3p to Strengthen HCC Cells Invasion and Migration. Oncotarget 7 (30), 47593–47608. 10.18632/oncotarget.10250 27351280 PMC5216964

[B208] Xu R. Greening D. W. Zhu H. J. Takahashi N. Simpson R. J. (2016). Extracellular Vesicle Isolation and Characterization: toward Clinical Application. J. Clin. Invest. 126 (4), 1152–1162. 10.1172/JCI81129 27035807 PMC4811150

[B209] Yang J. Kantrow S. Sai J. Hawkins O. E. Boothby M. Ayers G. D. (2012). INK4a/ARF [corrected] Inactivation with Activation of the NF-κB/IL-6 Pathway Is Sufficient to Drive the Development and Growth of Angiosarcoma. Cancer Res. 72 (18), 4682–4695. 10.1158/0008-5472.CAN-12-0440 22836752 PMC3459578

[B210] Yang J. Meng X. Pan J. Jiang N. Zhou C. Wu Z. (2018). CRISPR/Cas9-mediated Noncoding RNA Editing in Human Cancers. RNA Biol. 15 (1), 35–43. 10.1080/15476286.2017.1391443 29028415 PMC5785983

[B211] Yang J. J. Tao H. Li J. (2014). Hedgehog Signaling Pathway as Key Player in Liver Fibrosis: New Insights and Perspectives. Expert Opin. Ther. Targets 18 (9), 1011–1021. 10.1517/14728222.2014.927443 24935558

[B212] Yang J. J. Yang Y. Zhang C. Li J. Yang Y. (2020a). Epigenetic Silencing of LncRNA ANRIL Enhances Liver Fibrosis and HSC Activation through Activating AMPK Pathway. J. Cel Mol Med 24 (4), 2677–2687. 10.1111/jcmm.14987 PMC7028869 31961061

[B213] Yang L. Fu W. L. Zhu Y. Wang X. G. (2020b). Tβ4 Suppresses lincRNA-P21-Mediated Hepatic Apoptosis and Fibrosis by Inhibiting PI3K-AKT-NF-Κb Pathway. Gene 758, 144946. 10.1016/j.gene.2020.144946 32649978

[B214] Yang X. Xie Z. Lei X. Gan R. (2020c). Long Non-coding RNA GAS5 in Human Cancer. Oncol. Lett. 20 (3), 2587–2594. 10.3892/ol.2020.11809 32782576 PMC7400976

[B215] Yang Z. Jiang S. Shang J. Jiang Y. Dai Y. Xu B. (2019). LncRNA: Shedding Light on Mechanisms and Opportunities in Fibrosis and Aging. Ageing Res. Rev. 52, 17–31. 10.1016/j.arr.2019.04.001 30954650

[B216] Ye J. Lin Y. Yu Y. Sun D. (2020). LncRNA NEAT1/microRNA-129-5p/SOCS2 axis Regulates Liver Fibrosis in Alcoholic Steatohepatitis. J. Transl Med. 18 (1), 445. 10.1186/s12967-020-02577-5 33228663 PMC7686721

[B217] Yin C. Evason K. J. Asahina K. Stainier D. Y. (2013). Hepatic Stellate Cells in Liver Development, Regeneration, and Cancer. J. Clin. Invest. 123 (5), 1902–1910. 10.1172/JCI66369 23635788 PMC3635734

[B218] Yoon J. H. Abdelmohsen K. Srikantan S. Yang X. Martindale J. L. De S. (2012a). LincRNA-p21 Suppresses Target mRNA Translation. Mol. Cel 47 (4), 648–655. 10.1016/j.molcel.2012.06.027 PMC3509343 22841487

[B219] Yoon J. H. Srikantan S. Gorospe M. (2012b). MS2-TRAP (MS2-tagged RNA Affinity Purification): Tagging RNA to Identify Associated miRNAs. Methods 58 (2), 81–87. 10.1016/j.ymeth.2012.07.004 22813890 PMC3493847

[B220] Yoshimura H. Matsuda Y. Yamamoto M. Kamiya S. Ishiwata T. (2018). Expression and Role of Long Non-coding RNA H19 in Carcinogenesis. Front. Biosci. (Landmark Ed. 23, 614–625. 10.2741/4608 28930564

[B221] Young T. L. Matsuda T. Cepko C. L. (2005). The Noncoding RNA Taurine Upregulated Gene 1 Is Required for Differentiation of the Murine Retina. Curr. Biol. 15 (6), 501–512. 10.1016/j.cub.2005.02.027 15797018

[B222] Yu F. Dong P. Mao Y. Zhao B. Huang Z. Zheng J. (2019). Loss of lncRNA-SNHG7 Promotes the Suppression of Hepatic Stellate Cell Activation via miR-378a-3p and DVL2. Mol. Ther. Nucleic Acids 17, 235–244. 10.1016/j.omtn.2019.05.026 31272073 PMC6610663

[B223] Yu F. Geng W. Dong P. Huang Z. Zheng J. (2018). LncRNA-MEG3 Inhibits Activation of Hepatic Stellate Cells through SMO Protein and miR-212. Cel Death Dis 9 (10), 1014. 10.1038/s41419-018-1068-x PMC6170498 30282972

[B224] Yu F. Guo Y. Chen B. Shi L. Dong P. Zhou M. (2017a). LincRNA-p21 Inhibits the Wnt/β-Catenin Pathway in Activated Hepatic Stellate Cells via Sponging MicroRNA-17-5p. Cel Physiol Biochem 41 (5), 1970–1980. 10.1159/000472410 28391277

[B225] Yu F. Jiang Z. Chen B. Dong P. Zheng J. (2017b). NEAT1 Accelerates the Progression of Liver Fibrosis via Regulation of microRNA-122 and Kruppel-like Factor 6. J. Mol. Med. (Berl) 95 (11), 1191–1202. 10.1007/s00109-017-1586-5 28864835

[B226] Yu F. Lu Z. Cai J. Huang K. Chen B. Li G. (2015a). MALAT1 Functions as a Competing Endogenous RNA to Mediate Rac1 Expression by Sequestering miR-101b in Liver Fibrosis. Cell Cycle 14 (24), 3885–3896. 10.1080/15384101.2015.1120917 26697839 PMC4825734

[B227] Yu F. Lu Z. Chen B. Dong P. Zheng J. (2016). Identification of a Novel lincRNA-P21-miR-181b-PTEN Signaling Cascade in Liver Fibrosis. Mediators Inflamm. 2016, 9856538. 10.1155/2016/9856538 27610008 PMC5004029

[B228] Yu F. Zheng J. Mao Y. Dong P. Lu Z. Li G. (2015b). Long Non-coding RNA Growth Arrest-specific Transcript 5 (GAS5) Inhibits Liver Fibrogenesis through a Mechanism of Competing Endogenous RNA. J. Biol. Chem. 290 (47), 28286–28298. 10.1074/jbc.M115.683813 26446789 PMC4653684

[B229] Yu F. Zhou G. Huang K. Fan X. Li G. Chen B. (2017c). Serum lincRNA-P21 as a Potential Biomarker of Liver Fibrosis in Chronic Hepatitis B Patients. J. Viral Hepat. 24 (7), 580–588. 10.1111/jvh.12680 28107589

[B230] Yu L. Wang L. Wu X. Yi H. (2021). RSPO4-CRISPR Alleviates Liver Injury and Restores Gut Microbiota in a Rat Model of Liver Fibrosis. Commun. Biol. 4 (1), 230. 10.1038/s42003-021-01747-5 33603089 PMC7893072

[B231] Zhang B. Arun G. Mao Y. S. Lazar Z. Hung G. Bhattacharjee G. (2012). The lncRNA Malat1 Is Dispensable for Mouse Development but its Transcription Plays a Cis-Regulatory Role in the Adult. Cel Rep 2 (1), 111–123. 10.1016/j.celrep.2012.06.003 PMC3408587 22840402

[B232] Zhang C. Y. Yuan W. G. He P. Lei J. H. Wang C. X. (2016a). Liver Fibrosis and Hepatic Stellate Cells: Etiology, Pathological Hallmarks and Therapeutic Targets. World J. Gastroenterol. 22 (48), 10512–10522. 10.3748/wjg.v22.i48.10512 28082803 PMC5192262

[B233] Zhang D. M. Lin Z. Y. Yang Z. H. Wang Y. Y. Wan D. Zhong J. L. (2017a). IncRNA H19 Promotes Tongue Squamous Cell Carcinoma Progression through Beta-catenin/GSK3beta/EMT Signaling via Association with EZH2. Am. J. Transl Res. 9 (7), 3474–3486. 28804564 PMC5527262

[B234] Zhang H. Li W. Gu W. Yan Y. Yao X. Zheng J. (2019a). MALAT1 Accelerates the Development and Progression of Renal Cell Carcinoma by Decreasing the Expression of miR-203 and Promoting the Expression of BIRC5. Cel Prolif 52 (5), e12640. 10.1111/cpr.12640 PMC6797509 31250518

[B235] Zhang K. Han X. Zhang Z. Zheng L. Hu Z. Yao Q. (2017b). The Liver-Enriched Lnc-LFAR1 Promotes Liver Fibrosis by Activating TGFβ and Notch Pathways. Nat. Commun. 8 (1), 144. 10.1038/s41467-017-00204-4 28747678 PMC5529527

[B236] Zhang K. Shi Z. Zhang M. Dong X. Zheng L. Li G. (2020a). Silencing lncRNA Lfar1 Alleviates the Classical Activation and Pyoptosis of Macrophage in Hepatic Fibrosis. Cel Death Dis 11 (2), 132. 10.1038/s41419-020-2323-5 PMC7028920 32071306

[B237] Zhang K. Shi Z. M. Chang Y. N. Hu Z. M. Qi H. X. Hong W. (2014). The Ways of Action of Long Non-coding RNAs in Cytoplasm and Nucleus. Gene 547 (1), 1–9. 10.1016/j.gene.2014.06.043 24967943

[B238] Zhang K. Zhang M. Yao Q. Han X. Zhao Y. Zheng L. (2019b). The Hepatocyte-Specifically Expressed Lnc-HSER Alleviates Hepatic Fibrosis by Inhibiting Hepatocyte Apoptosis and Epithelial-Mesenchymal Transition. Theranostics 9 (25), 7566–7582. 10.7150/thno.36942 31695787 PMC6831459

[B239] Zhang Q. Geng P. L. Yin P. Wang X. L. Jia J. P. Yao J. (2013). Down-regulation of Long Non-coding RNA TUG1 Inhibits Osteosarcoma Cell Proliferation and Promotes Apoptosis. Asian Pac. J. Cancer Prev. 14 (4), 2311–2315. 10.7314/apjcp.2013.14.4.2311 23725133

[B240] Zhang R. Huang X. Q. Jiang Y. Y. Li N. Wang J. Chen S. Y. (2020b). LncRNA TUG1 Regulates Autophagy-Mediated Endothelial-Mesenchymal Transition of Liver Sinusoidal Endothelial Cells by Sponging miR-142-3p. Am. J. Transl Res. 12 (3), 758–772. 32269710 PMC7137070

[B241] Zhang T. Hu J. Wang X. Zhao X. Li Z. Niu J. (2019c). MicroRNA-378 Promotes Hepatic Inflammation and Fibrosis via Modulation of the NF-Κb-Tnfα Pathway. J. Hepatol. 70 (1), 87–96. 10.1016/j.jhep.2018.08.026 30218679 PMC6554744

[B242] Zhang X. Rice K. Wang Y. Chen W. Zhong Y. Nakayama Y. (2010). Maternally Expressed Gene 3 (MEG3) Noncoding Ribonucleic Acid: Isoform Structure, Expression, and Functions. Endocrinology 151 (3), 939–947. 10.1210/en.2009-0657 20032057 PMC2840681

[B243] Zhang Y. Liu C. Barbier O. Smalling R. Tsuchiya H. Lee S. (2016b). Bcl2 Is a Critical Regulator of Bile Acid Homeostasis by Dictating Shp and lncRNA H19 Function. Sci. Rep. 6, 20559. 10.1038/srep20559 26838806 PMC4738356

[B244] Zhang Y. F. Li C. S. Zhou Y. Lu X. H. (2020c). Propofol facilitates cisplatin sensitivity via lncRNA MALAT1/miR-30e/ATG5 axis through suppressing autophagy in gastric cancer. Life Sci. 244, 117280. 10.1016/j.lfs.2020.117280 31926239

[B245] Zhang Y. H. Liu J. T. Wen B. Y. Liu N. (2009). Mechanisms of Inhibiting Proliferation of Vascular Smooth Muscle Cells by Serum of Rats Treated with Dahuang Zhechong Pill. J. Ethnopharmacol 124 (1), 125–129. 10.1016/j.jep.2009.04.012 19527826

[B246] Zhang Z. Qian W. Wang S. Ji D. Wang Q. Li J. (2018). Analysis of lncRNA-Associated ceRNA Network Reveals Potential lncRNA Biomarkers in Human Colon Adenocarcinoma. Cel Physiol Biochem 49 (5), 1778–1791. 10.1159/000493623 30231249

[B247] Zhao J. Dahle D. Zhou Y. Zhang X. Klibanski A. (2005). Hypermethylation of the Promoter Region Is Associated with the Loss of MEG3 Gene Expression in Human Pituitary Tumors. J. Clin. Endocrinol. Metab. 90 (4), 2179–2186. 10.1210/jc.2004-1848 15644399

[B248] Zhao J. Han M. Zhou L. Liang P. Wang Y. Feng S. (2020). TAF and TDF Attenuate Liver Fibrosis through NS5ATP9, TGFβ1/Smad3, and NF-Κb/nlrp3 Inflammasome Signaling Pathways. Hepatol. Int. 14 (1), 145–160. 10.1007/s12072-019-09997-6 31758498

[B249] Zhao Y. Hu X. Liu Y. Dong S. Wen Z. He W. (2017). ROS Signaling under Metabolic Stress: Cross-Talk between AMPK and AKT Pathway. Mol. Cancer 16 (1), 79. 10.1186/s12943-017-0648-1 28407774 PMC5390360

[B250] Zhao Z. Lin C. Y. Cheng K. (2019). siRNA- and miRNA-Based Therapeutics for Liver Fibrosis. Transl Res. 214, 17–29. 10.1016/j.trsl.2019.07.007 31476281 PMC6848786

[B251] Zheng J. Dong P. Mao Y. Chen S. Wu X. Li G. (2015). lincRNA-p21 Inhibits Hepatic Stellate Cell Activation and Liver Fibrogenesis via P21. FEBS J. 282 (24), 4810–4821. 10.1111/febs.13544 26433205

[B252] Zheng J. Mao Y. Dong P. Huang Z. Yu F. (2019). Long Noncoding RNA HOTTIP Mediates SRF Expression through Sponging miR-150 in Hepatic Stellate Cells. J. Cel Mol Med 23 (2), 1572–1580. 10.1111/jcmm.14068 PMC6349348 30548190

[B253] Zhong Q. Wang Z. Liao X. Wu R. Guo X. (2020). LncRNA GAS5/miR-4465 axis R-egulates the M-alignant P-otential of N-asopharyngeal C-arcinoma by T-argeting COX2. Cell Cycle 19 (22), 3004–3017. 10.1080/15384101.2020.1816280 33092435 PMC7714527

[B254] Zhou B. Yuan W. Li X. (2018). LncRNA Gm5091 Alleviates Alcoholic Hepatic Fibrosis by Sponging miR-27b/23b/24 in Mice. Cell Biol Int 42 (10), 1330–1339. 10.1002/cbin.11021 29935035

[B255] Zhou L. Liu Y. (2015). Wnt/β-catenin Signalling and Podocyte Dysfunction in Proteinuric Kidney Disease. Nat. Rev. Nephrol. 11 (9), 535–545. 10.1038/nrneph.2015.88 26055352 PMC4869701

[B256] Zhou W. Ye X. L. Xu J. Cao M. G. Fang Z. Y. Li L. Y. (2017). The lncRNA H19 Mediates Breast Cancer Cell Plasticity during EMT and MET Plasticity by Differentially Sponging miR-200b/c and Let-7b. Sci. Signal. 10 (483), eaak9557. 10.1126/scisignal.aak9557 28611183

[B257] Zhou Y. Zhang X. Klibanski A. (2012). MEG3 Noncoding RNA: a Tumor Suppressor. J. Mol. Endocrinol. 48 (3), R45–R53. 10.1530/JME-12-0008 22393162 PMC3738193

[B258] Zhu C. Tabas I. Schwabe R. F. Pajvani U. B. (2021). Maladaptive Regeneration - the Reawakening of Developmental Pathways in NASH and Fibrosis. Nat. Rev. Gastroenterol. Hepatol. 18 (2), 131–142. 10.1038/s41575-020-00365-6 33051603 PMC7854502

[B259] Zhu J. Luo Z. Pan Y. Zheng W. Li W. Zhang Z. (2019). H19/miR-148a/USP4 axis Facilitates Liver Fibrosis by Enhancing TGF-β Signaling in Both Hepatic Stellate Cells and Hepatocytes. J. Cel Physiol 234 (6), 9698–9710. 10.1002/jcp.27656 30362572

[B260] Zuo X.-x. Yang Y. Zhang Y. Zhang Z.-g. Wang X.-f. Shi Y.-g. (2019). Platelets Promote Breast Cancer Cell MCF-7 Metastasis by Direct Interaction: Surface Integrin α2β1-contacting-mediated Activation of Wnt-β-Catenin Pathway. Cell Commun Signal 17 (1), 142. 10.1186/s12964-019-0464-x 31699102 PMC6836423

